# Targeting neuronal lysosomal dysfunction caused by β-glucocerebrosidase deficiency with an enzyme-based brain shuttle construct

**DOI:** 10.1038/s41467-023-37632-4

**Published:** 2023-04-12

**Authors:** Alexandra Gehrlein, Vinod Udayar, Nadia Anastasi, Martino L. Morella, Iris Ruf, Doris Brugger, Sophia von der Mark, Ralf Thoma, Arne Rufer, Dominik Heer, Nina Pfahler, Anton Jochner, Jens Niewoehner, Luise Wolf, Matthias Fueth, Martin Ebeling, Roberto Villaseñor, Yanping Zhu, Matthew C. Deen, Xiaoyang Shan, Zahra Ehsaei, Verdon Taylor, Ellen Sidransky, David J. Vocadlo, Per-Ola Freskgård, Ravi Jagasia

**Affiliations:** 1 Roche Pharma Research and Early Development, Neuroscience and Rare Diseases Discovery and Translational Area, Roche Innovation Center Basel, F. Hoffmann-La Roche Ltd, Basel, Switzerland; 2 Department of Anatomy and Neurosciences, Amsterdam University Medical Center | VUmc, Amsterdam, Netherlands; 3 Roche Pharma Research and Early Development, Therapeutic Modalities, Lead Discovery, Roche Innovation Center Basel, F. Hoffmann-La Roche Ltd, Basel, Switzerland; 4 Interfaculty Institute of Biochemistry & Structural Biology Biochemistry (IFIB), Eberhard Karls University of Tübingen, Tübingen, Germany; 5 Roche Pharma Research and Early Development, Therapeutic Modalities Large Molecule Research, Roche Innovation Center Munich, Roche Diagnostics GmbH, Penzberg, Germany; 6 Roche Pharma Research and Early Development, Data & Analytics, Roche Innovation Center Basel, F. Hoffmann-La Roche Ltd, Basel, Switzerland; 7 Roche Pharma Research and Early Development, Pharmaceutical Science, Roche Innovation Center Basel, F. Hoffmann-La Roche Ltd, Basel, Switzerland; 8 Department of Chemistry, Simon Fraser University, Burnaby, BC V5A 1S6 Canada; 9 Department of Biomedicine, University of Basel, Basel, Switzerland; 10 Molecular Neurogenetics Section, National Human Genome Research Institute, Bethesda, MD USA; 11 Department of Molecular Biology and Biochemistry, Simon Fraser University, Burnaby, BC V5A 1S6 Canada; 12 BioArctic AB, Stockholm, Sweden

**Keywords:** Neurodegeneration, Lysosomes, Protein delivery

## Abstract

Mutations in glucocerebrosidase cause the lysosomal storage disorder Gaucher’s disease and are the most common risk factor for Parkinson’s disease. Therapies to restore the enzyme’s function in the brain hold great promise for treating the neurological implications. Thus, we developed blood-brain barrier penetrant therapeutic molecules by fusing transferrin receptor-binding moieties to β-glucocerebrosidase (referred to as GCase-BS). We demonstrate that these fusion proteins show significantly increased uptake and lysosomal efficiency compared to the enzyme alone. In a cellular disease model, GCase-BS rapidly rescues the lysosomal proteome and lipid accumulations beyond known substrates. In a mouse disease model, intravenous injection of GCase-BS leads to a sustained reduction of glucosylsphingosine and can lower neurofilament-light chain plasma levels. Collectively, these findings demonstrate the potential of GCase-BS for treating *GBA1*-associated lysosomal dysfunction, provide insight into candidate biomarkers, and may ultimately open a promising treatment paradigm for lysosomal storage diseases extending beyond the central nervous system.

## Abstract (web summary)

Mutations in glucocerebrosidase (GCase) cause the lysosomal storage disorder Gaucher’s disease and are the most common risk factor for Parkinson’s disease. Using a fusion protein comprising GCase and a transferrin receptor antibody fragment, the authors show that the transferrin receptor pathway can be therapeutically exploited to both pass the blood-brain barrier and efficiently target lysosomal GCase deficiency.

## Introduction

Lysosomes host more than 60 soluble lysosomal hydrolases and accessory proteins, as well as over 120 lysosomal membrane proteins and transitory protein residents^1^. Dysfunction in some of these proteins lead to lysosomal storage disorders (LSDs), which collectively have a relatively high incidence in the general population: more than 1:5000 live births are affected by a LSD^2^. Notable among these lysosomal enzymes is β-glucocerebrosidase (GCase), which is encoded by *GBA1*, and is responsible for the hydrolysis of glucosylceramide (GlcCer). Loss of GCase enzymatic activity leads to an accumulation of GlcCer which subsequently leads to a secondary accumulation of its deacylated form glucosylsphingosine (GlcSph) in the lysosome^3–5^.

Mutations in *GBA1* cause Gaucher disease (GD) with varying disease severity^6^. Neuronopathic GD type 2 results from severe or null mutations in *GBA1* and is ultimately lethal. Currently, there is no medication that alters its disease trajectory^7–10^. While often debilitating, chronic neuronopathic or GD type 3 has varying degrees of neurological manifestations, but patients survive infancy and can present late^11^. Both homozygous and heterozygous carriers of mutant *GBA1* alleles are at increased risk for sporadic and complex neurodegenerative diseases including Parkinson’s disease (PD) and dementia with Lewy Bodies (DLB). Here too, there is no disease-modifying therapy that slows the trajectory of the disease. *GBA1*-associated PD, while neuropathologically indistinguishable from sporadic PD, is often associated with an earlier disease onset, more pronounced non-motor symptoms and a faster disease progression^12,13^. It is hoped that treatment paradigms targeting impaired GCase to restore its intracellular lysosomal function will prove beneficial for disorders ranging from neuronopathic GD to neurodegenerative diseases such as PD and DLB.

GD- and PD risk-associated pathologic mutations in *GBA*1, for example, L444P (p.L483P) and N370S (p.N409P) lead to the production of misfolded GCase with significantly reduced activity, in the range of 10 to 20% of normal^14^. The consequential accumulation of glycosphingolipids is a key pathological event in GD and may be a triggering event for the neurodegeneration associated with PD^15–17^. Thus, it is very likely, given the shared genetics, that increasing GCase lysosomal activity in the brain would be a viable therapy to restore lysosomal homeostasis in both neuronopathic GD and *GBA1*-associated neurodegenerative diseases.

Currently available treatments for GD include substrate reduction therapy using small molecules or enzyme replacement therapy (ERT). Both, however, fail to target GCase deficiency within the central nervous system (CNS). To overcome these limitations, we exploited the known ability of transferrin protein to cross the blood-brain barrier (BBB) through binding to the transferrin receptor (TfR), which transports the iron-binding protein transferrin into the brain^18,19^. Moreover, we reasoned that hijacking this TfR-mediated pathway could also lead to increased lysosomal localisation of a cargo protein such as GCase^20,21^. With this in mind, we fused a fragment of a TfR antibody to recombinant human or murine GCase to generate a fusion protein we termed the GCase Brain Shuttle (GCase-BS). We assessed its efficacy in correcting *GBA1*-associated molecular changes in vitro and in vivo. We demonstrated that GCase-BS TfR-binders not only mediate successful transcytosis of GCase across endothelial cells of the BBB, but they are significantly more efficient than conventional ERT using recombinant GCase in terms of delivering enzyme to the lysosomal compartment and driving the hydrolysis of accumulated neurotoxic GlcSph and GlcCer in multiple neuronal models. Our results also uncover GD-associated lysosomal protein- and lipid defects that are rapidly corrected upon delivery of GCase-BS. Our data provide a pre-clinical proof of concept support for the use of GCase-BS for the treatment of *GBA1-*associated accumulation of neurotoxic GlcSph and GlcCer. Furthermore, this work provides insights into putative GD-associated lysosomal biomarkers downstream of GCase and highlights areas requiring further optimisation. Given that the TfR targeting is so efficacious, it is conceivable that this approach may extend to multiple LSDs even in peripheral tissue that are not well targeted to date.

## Results

### Purified GCase-BS molecules are functional with respects to enzymatic activity, TfR binding and stability

**Fig. 1:**
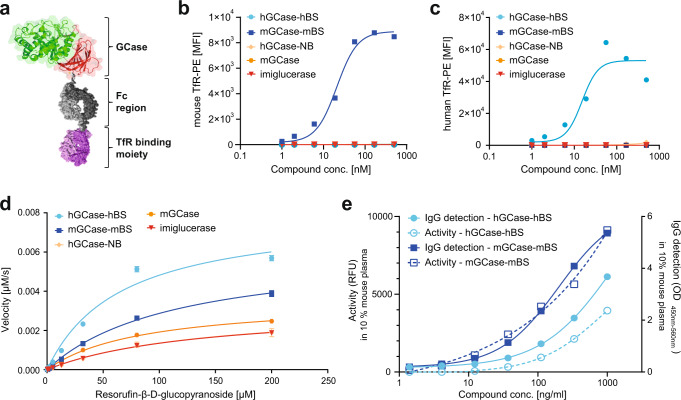
Purified GCase-BS molecules are fully functional. **a** Schematic of GCase-BS depicting different moieties: GCase domain II is shown in red, domain III in green, domain I is hidden behind domain III in this view^23^. The human IgG1 Fc part of the Brain Shuttle is shown in grey, the TfR binding Fab is depicted in purple. **b** Assessment and specificity of murine TfR binding of the various GCase(-BS) molecules by FACS analysis of mTfR-expressing cells. Representative data of 2 biological replicates. **c** Assessment and specificity of human TfR binding of the various GCase(-BS) molecules by FACS analysis of hTfR-expressing cells. Representative data of 2 biological replicates. **d** Enzymatic activity of various GCase(-BS) molecules at an enzyme concentration of 25 nM (Michaelis-Menten kinetics). Activity was measured over time using different concentrations of resorufin-β-glucopyranoside. *n* = 3 independent measurements. **e** Enzymatic activity and IgG levels measured for both mGCase-mBS and hGCase-hBS after 15 min incubation in 10% mouse plasma. *n* = 2 independent measurements. Source data are provided as a Source Data file.

We designed GCase-BS molecules in which one chain of a human IgG1 Fc portion was fused to the C-terminus of GCase whereas the other chain of the Fc portion was fused N-terminally to an anti-mouse or anti-human TfR-binding Fab. These fusion constructs, referred to as mGCase-mBS or hGCase-hBS, respectively, were designed using knob-into-hole technology, which was used to enable monovalent binding to TfR^22^; (Fig. 1a). The colour coding depicts different domains of the enzyme: domain II is shown in red, domain III in green, domaín I is hidden behind domain III in this view^23^.

We generated a variety of insect cell (S2)-derived constructs and analysed the purified molecules to assess their TfR binding and enzymatic properties (Fig. 1b–d, Supplementary Table 1). Binding to mouse or human TfR was evaluated by FACS performed using suitable TfR-expressing cell lines, and the EC_50_ values of the selected fusion constructs were 21 nM for mGCase-mBS or 15 nM for hGCase-hBS, respectively (Fig. 1b–c). We benchmarked the enzymatic properties of all molecules described in the manuscript to the conventional ERT molecule imiglucerase (commercially available as Cerezyme) (see Supplementary Table 1). There was a tendency for the Brain Shuttle constructs to be more efficient in terms of the ratio of turnover to affinity to resorufin-β-D-glucopyranoside. The enzymatic properties of mGCase and TfR non-binder (GCase-NB) are very similar to imiglucerase (Fig. 1d and Supplementary Table 1). Incubation of the produced murine and human GCase-BS constructs in 10% mouse plasma for a period of 15 min at physiological pH revealed that both stability and enzymatic activity were maintained (Fig. 1e).

We furthermore analysed the biochemical hydrolysis of GlcCer or GlcSph using imiglucerase or GCase-BS. For both glycolipids, 30 min of incubation with the enzyme yielded the respective lipid product (ceramide or sphingosine) whilst without enzyme no product was detectable (Supplementary Fig. 1). These findings suggest that recombinant GCase molecules can hydrolyse both substrates, going in line with previous literature^24,25^.

### The TfR-binding module improves lysosomal targeting and substrate reduction in vitro

**Fig. 2:**
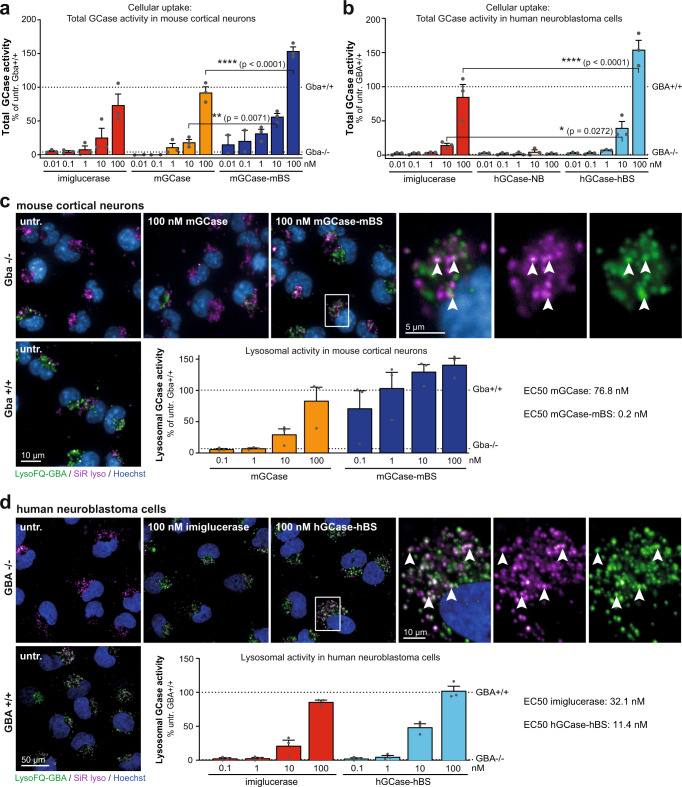
The Brain Shuttle module improves cellular uptake and lysosomal activity in vitro. **a** Total GCase activity in mouse cortical neurons as a measure of cellular uptake after 2 h of treatment with imiglucerase, mGCase or mGCase-mBS. Data was normalised to *Gba* + /*+* cells. *n* = 3 (for mGCase and mGCase-mBS, the two lowest concentrations have only been repeated twice). **b** Total GCase activity in H4 cells as a measure of cellular uptake after 2 h of treatment with imiglucerase, hGCase-hBS-NB or hGCase-hBS. Data was normalised to *GBA* + / + cells. *n* = 3. **c** Live imaging of GCase activity (LysoFQ-GBA) and lysosomes (SiR lyso) and quantification of colocalising signal in mouse cortical neurons. Data was normalised to *Gba* + /*+* cells. *n* = 3 (4–9 fields analysed per plate). **d** Live imaging of GCase activity (LysoFQ-GBA) and lysosomes (SiR lyso) and quantification of colocalising signal in H4 cells. Data was normalised to *GBA* + / + cells. *n* = 3 (8–16 fields analysed per plate). Bar graphs represent group means + SEM. Each data point represents an independent measurement. Activity data were analysed by two-way ANOVA (Tukey’s multiple comparisons test). *p < 0.05; **p < 0.01; ***p < 0.001; ****p < 0.0001. *n* = number of independent measurements. Source data are provided as a Source Data file.

**Fig. 3:**
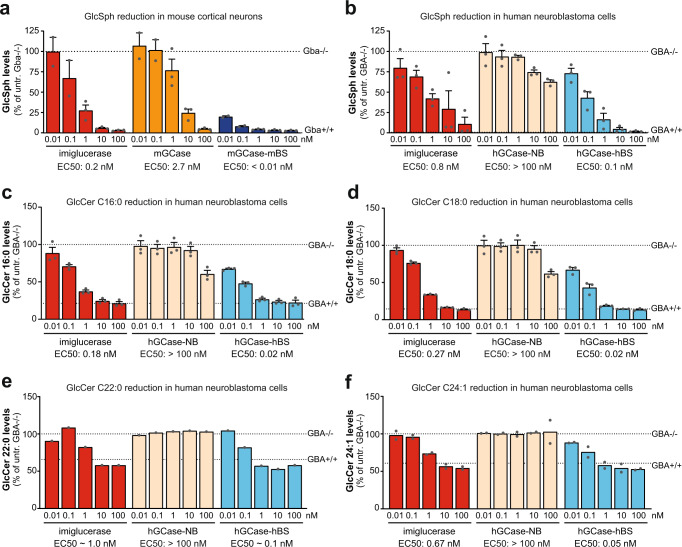
The Brain Shuttle module improves the breakdown of GlcSph and multiple GlcCer isomers in vitro. **a** GlcSph measurement in mouse cortical neurons as a measure of efficacy after 48 h of treatment with imiglucerase, mGCase or mGCase-mBS. Data was normalised to *Gba*−/− cells. *n* = 3. **b** GlcSph measurement in H4 cells as a measure of catalytic efficacy after 48 h of treatment with imiglucerase, hGCase-NB or hGCase-hBS. Data was normalised to *GBA*−/− cells. *n* = 3. **c** GlcCer C16:0 measurement in H4 cells as a measure of catalytic efficacy after 48 h of treatment with imiglucerase, hGCase-NB or hGCase-hBS. *n* = 3. **d** GlcCer C18:0 measurement in H4 cells as a measure of catalytic efficacy after 48 h of treatment with imiglucerase, hGCase-NB or hGCase-hBS. *n* = 3. **e** GlcCer C22:0 measurement in H4 cells as a measure of catalytic efficacy after 48 h of treatment with imiglucerase, hGCase-NB or hGCase-hBS. *n* = 1. **f** GlcCer C24:1 measurement in H4 cells as a measure of catalytic efficacy after 48 h of treatment with imiglucerase, hGCase-NB or hGCase-hBS. *n* = 2. Bar graphs represent group means + SEM. Each data point represents an independent measurement. *n* = number of independent measurements. Source data are provided as a Source Data file.

Since we aimed to develop a molecule to target neuronal dysfunction associated with GD, we sought to investigate the effects of the TfR-binding module on cellular uptake and lysosomal efficacy in neuronal cells. To this end, we used a variety of GCase-deficient cell lines: immortalised mouse cortical neurons from embryonic null allele *Gba−/−* mice^26^, human pluripotent stem cell-derived neurons or human neuroblastoma cells (H4 cells) in which *GBA1* was deleted (*GBA−/−*)^27^ or primary murine neurons harbouring a human *Gba1 h*omozygous mutation (*Gba D409V/D409V*)^28^. Both murine and human cell lines exhibit reduced basal GCase activity as well as significantly elevated lysosomal glycolipid levels compared to respective WT cells^26^ (and Fig. 2 and Fig. 3: *GBA* + */* + vs. *GBA−/−;* Supplementary Fig. 2*Gba* + */+* vs. *Gba D409V/D409V*).

In cellular uptake experiments, a 2 h treatment of *Gba−/−* immortalised mouse cortical neurons with mGCase-mBS led to a dose-dependent increase in total GCase activity which normalised function between 10–100 nM with respect to WT levels. Compared to mGCase or imiglucerase, mGCase-mBS showed a clear increase in cellular uptake most notable at the highest concentration tested (Fig. 2a). Consistently, in *GBA−/−* H4 cells, a 2 h treatment with hGCase-hBS increased total GCase activity up to 3-fold more than imiglucerase, suggesting that uptake of hGCase-hBS is superior to GCase alone (Fig. 2b). Interestingly, there was less uptake of hGCase fused to a TfR non-binder (Fig. 2b, hGCase-NB), illustrating that this construct had reduced propensity for cellular uptake compared to the commercially available enzyme. This could be explained by the size difference since basal biochemical properties cannot account for it.

We next exploited a GCase-specific fluorescence-quenched substrate LysoFQ-GBA^29^ to measure increase in lysosomal GCase activity in neurons. Cell imaging revealed that treating *Gba*-deficient immortalised murine cortical neurons for 2 h with mGCase or mGCase-mBS could restore lysosomal GCase activity to WT levels. We found that LysoFQ-GBA signal colocalised with the lysosomal probe SiR-lysosome and quantification of GCase activity showed that mGCase-mBS (EC_50_ = 0.2 nM) was >100-times more effective than mGCase (EC_50_ = 76.8 nM) in restoring lysosomal GCase activity (Fig. 2c). In primary neurons derived from *Gba1 D409V/D409V* mice, GCase activity was ~7-fold higher for mGCase-mBS as compared to an equimolar dose of mGCase (32 nM, Supplementary Fig. 2a). In *GBA−/−* H4 cells, a 2 h treatment with imiglucerase or hGCase-hBS led to a significant increase in lysosomal GCase activity. Quantification of the LysoFQ-GBA signal revealed that hGCase-hBS (EC_50_ = 11.4 nM) was 3-fold more efficient at increasing lysosomal GCase activity as compared to imiglucerase (EC_50_ = 32.1 nM) (Fig. 2d). These data illustrate that for both the murine and human constructs, GCase alone is taken up less efficiently into lysosomes than our GCase-BS constructs, which actively engage with the TfR.

Upon 48 h incubation with the respective molecule we found that, in both mouse and human cellular systems, lysosomal GlcSph was more efficently reduced when GCase was fused to a TfR-binding moiety. Comparison of EC_50_ values revealed that mGCase-mBS was >100-fold more efficacious than mGCase alone and hGCase-hBS was ~8-fold more efficacious than imiglucerase (Fig. 3a–b). We furthermore analysed the reduction of several GlcCer isomers in *GBA−/−* H4 cells and could show that hGCase-hBS also reduced GlcCer more efficiently. The comparison of EC_50_ values to imiglucerase revealed that hGCase-hBS was ~10-fold more efficacious in reducing all GlcCer isoforms that were analysed (C16:0: EC_50_ imiglucerase = 0.18 nM vs. EC_50_ hGCase-hBS = 0.02 nM; C18:0: EC_50_ imiglucerase = 0.27 nM vs. EC_50_ hGCase-hBS = 0.02 nM; C22:0: EC_50_ imiglucerase ~1.0 nM vs. EC_50_ hGCase-hBS ~0.1 nM; C24:1: EC_50_ imiglucerase = 0.67 nM vs. EC_50_ hGCase-hBS = 0.05 nM; Fig. 3c–f). As a control, we showed that hGCase fused to a TfR non-binding module (hGCase-NB) resulted in limited substrate reduction, consistent with its inefficient cellular uptake (Fig. 2b and Fig. 3b–f).

Using immortalised mouse *Gba−/−* cortical neurons or *GBA−/−* H4 cells, we performed kinetic studies of GlcSph levels. Cells were incubated for 2 h with varying concentrations of mGCase-mBS and hGCase-hBS, respectively (Tmax), followed by washout, after which GlcSph levels were monitored over time (Supplementary Fig. 2b–c). In both experiments, GlcSph levels were halved within 6 h after washout and reached maximal efficacy by 24 h at all concentrations tested. Interestingly, a short incubation with 100 nM and 10 nM of GCase-BS resulted in sustained lipid reduction over the 72 h course of the experiment whilst the 1 nM incubation showed GlcSph levels starting to rebound back to those seen in the disease state. These data indicate that a 2 h incubation is sufficient to resolve substrate levels over an extended period of time, suggesting a mechanism driven by the maximum concentration of GCase in lysosomes. These data provided the foundation of the in vivo pharmacokinetics and -dynamics (PK/PD) studies.

We sought to verify our findings also in cell types expressing high levels of mannose receptor (MR), as imiglucerase was designed to target peripheral macrophages via terminal mannose^30,31^, and both H4 cells and neurons had no detectable MR protein levels (Supplementary Fig. 9). To this end, we used hiPSC-derived macrophages from a healthy donor (*GBA*+*/*+) or from a PD individual with a heterozygous *GBA1* mutation (*GBA N370S/*+). Nine days of treatment with 100 nM of hGCase-hBS led to a significant increase in total GCase activity (+13% in *GBA*+*/*+; +28% in *GBA N370S/*+; Supplementary Fig. 3a). Analysis of GlcSph levels showed that its levels were significantly elevated in the *GBA1 N370S/*+ line (+94%) and could be substantially reduced upon treatment with hGCase-hBS (79% reduction at 100 nM) (Supplementary Fig. 3b). In comparison to imiglucerase, hGCase-hBS was almost twice as efficacious in GlcSph reduction in macrophages (Supplementary Fig. 3c). Very similar results were obtained from hiPSC-derived microglia from a healthy donor (*GBA*+*/*+) or from a PD individual with *GBA1* genotype N370S/+ (*GBA1 N370S/*+). Upon nine days of treatment with hGCase-hBS, GCase activity was increased by 28% in the *GBA1 N370S/*+ line (Supplementary Fig. 3d). Compared to *GBA*+*/*+ cells, a significant accumulation of GlcSph was observed in the *GBA1 N370S/*+ line (+93%), which could be substantially lowered by hGCase-hBS (−42%) (Supplementary Fig. 3e). These data suggest that the TfR lysosomal routing pathway is conserved in macrophages and microglia and normalises enzyme activity and GlcSph levels lipids in a cellular model harbouring a GD/PD pathological mutation.

In human pluripotent stem cell-derived *GBA−/−* dopaminergic neurons^32^, treatment with 10 nM of hGCase-hBS efficiently normalised GlcSph levels (Supplementary Fig. 3f), suggesting that in human midbrain neurons, relevant for PD and GD, hGCase-hBS is efficacious at low concentrations.

### GCase-BS lysosomal mode of action in vitro

**Fig. 4:**
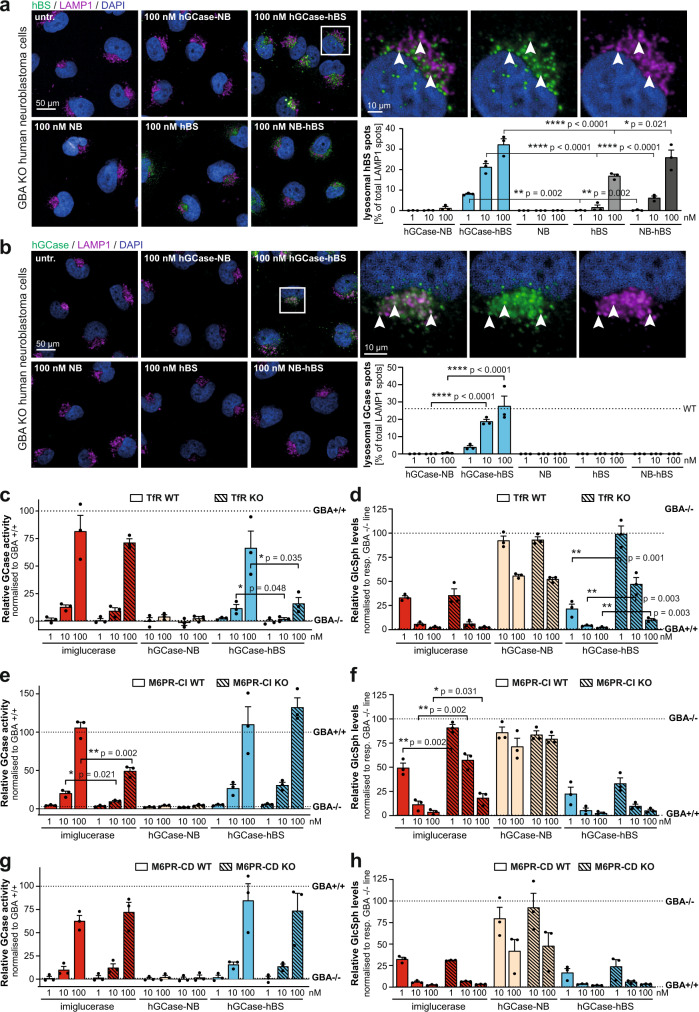
GCase-BS lysosomal mode of action in vitro. **a** Immunolabeling of hBS and colocalisation with LAMP1 upon acute treatment with various constructs. White arrows indicate some colocalising spots. Colocalising hBS spots were quantified and normalised to total amount of LAMP1 spots. *n* = 3. **b** Immunolabeling of hGCase and colocalisation with LAMP1 upon acute treatment with various constructs. White arrows indicate some colocalising spots. Colocalising GCase spots were quantified and normalised to total amount LAMP1 spots. *n* = 3. **c** Total GCase activity in GBA-deficient TfR WT and TfR KO neuroblastoma lines as a measure of cellular uptake after 2 h of treatment. Data was normalised to *GB*+/+ cells. *n* = 3. **d** GlcSph measurement in GBA-deficient TfR WT and TfR KO neuroblastoma lines upon 48 h of treatment. Data was normalised to respective *GBA*−/− cells. *n* = 3. **e** Total GCase activity in GBA-deficient M6PR-CI WT and M6PR-CI KO neuroblastoma lines as a measure of cellular uptake after 2 h of treatment. Data was normalised to *GBA*+/+ cells. *n* = 3. **f** GlcSph measurement in GBA-deficient M6PR-CI WT and M6PR-CI KO neuroblastoma lines upon 48 h of treatment. Data was normalised to respective *GBA*−/− cells. *n* = 3. **g** Total GCase activity in GBA-deficient M6PR-CD WT and M6PR-CD KO neuroblastoma lines as a measure of cellular uptake after 2 h of treatment. Data was normalised to *GB*+/+ cells. *n* = 3. **h** GlcSph measurement in GBA-deficient M6PR-CD WT and M6PR-CD KO neuroblastoma lines upon 48 h of treatment. Data was normalised to respective *GBA*−/− cells. *n* = 3. Bar graphs represent group means + SEM. Each data point represents an independent measurement. Data were analysed by Student’s two-tailed *t*-test comparing WT and KO of each receptor for each treatment. *p < 0.05; **p < 0.01; ***p < 0.001. If not stated otherwise, *n* = number of independent measurements. Source data are provided as a Source Data file.

It is well established that TfR binders transport therapeutic molecules across the BBB^21,33,34^. However, it is unclear which receptors are involved, or how the BS module of GCase-BS helps to facilitate cellular uptake and lysosomal targeting of GCase within the CNS. Inspired by the findings that the BS modules increased both lysosomal exposure and efficacy for hydrolysing GlcSph within lysosomes, we aimed to elucidate the underlying mechanisms in more detail. To this end, we compared the ability of four constructs to target the lysosome: (1) GCase attached to a TfR-binding molecule (hGCase-hBS), (2) GCase attached to a TfR-non binder (hGCase-NB), (3) an antibody cargo (NB-hBS) and (4) the BS (hBS) and control BS (NB) moieties alone. GCase-deficient H4 cells were incubated with the molecules for 2 h and the localisation of both the Brain Shuttle moiety and enzyme were monitored. We found that there was negligible cellular uptake of hGCase-NB and exposure to the lysosome, which we assessed based on little to no IgG or hGCase cellular immunoreactivity colocalising with lysosomal-associated membrane protein 1 (LAMP1) (Fig. 4a–b). This observation was in line with the previous findings that hGCase-NB only led to very limited cellular uptake and lysosomal GlcSph reduction (Fig. 2b and Fig. 3b). We also observed that 2 h treatment with hGCase-hBS led to colocalisation of both GCase and hBS with LAMP1 (approx. 30% colocalised spots at 100 nM). We observed considerably less colocalisation for NB-hBS or hBS alone (Fig. 4a–b), suggesting that efficient lysosomal targeting required a combination of both the BS module and its GCase cargo.

We next sought to determine which receptors are required in human neuronal cells for both cellular uptake and lysosomal targeting/efficacy of hGCase-hBS. In our experiments, we excluded the assumption that this was mediated by the MR since single cell analysis from brain tissue revealed that only microglia expressed reasonable levels^35^. In both H4 cells and human neurons, protein analysis revealed no expression of MR whilst mannose-6-phosphate receptor (M6PR) and TfR proteins were expressed (Supplementary Fig. 4 and 9). Thus, we generated several double knock-out (KO) H4 lines for GBA and either TfR, cation-dependent M6PR (M6PR-CD) or cation-independent M6PR (M6PR-CI) genes, yielding the cell lines GBA/TfR KO, GBA/M6PR-CD KO and GBA/M6PR-CI KO cells (Supplementary Fig. 4). After a 2 h treatment with imiglucerase, hGCase-NB, or hGCase-hBS we observed that absence of TfR resulted in marked reduction of cellular uptake of hGCase-hBS and significant impairment in the ability of the construct to reduce GlcSph. In contrast, for imiglucerase, cellular uptake and efficacy remained unaffected (Fig. 4c–d). However, using M6PR-CI KO cells, we found that both the uptake of enzyme and its ability to reduce GlcSph were significantly impacted for imiglucerase. Whilst imiglucerase is designed to target the mannose-receptor by terminal mannose groups, previous work had demonstrated that it can bind to M6PR despite the low levels of M6P present in its glycans^36^. In contrast, uptake and efficacy of hGCase-hBS were unaffected in M6PR-CI KO cells (Fig. 4e–f). Interestingly, we found that M6PR-CD played no role in neither cellular uptake nor lysosomal GlcSph reduction for any of the molecules (Fig. 4g–h).

Collectively, our data suggest that the hGCase-hBS construct predominantly accesses the lysosome by engagement and sorting through interaction with the TfR. We furthermore showed that even in myeloid cells with high MR expression, the TfR-binding moiety could improve uptake and lysosomal efficacy in comparison to imiglucerase.

### GCase-BS corrects lysosomal phenotypes

**Fig. 5:**
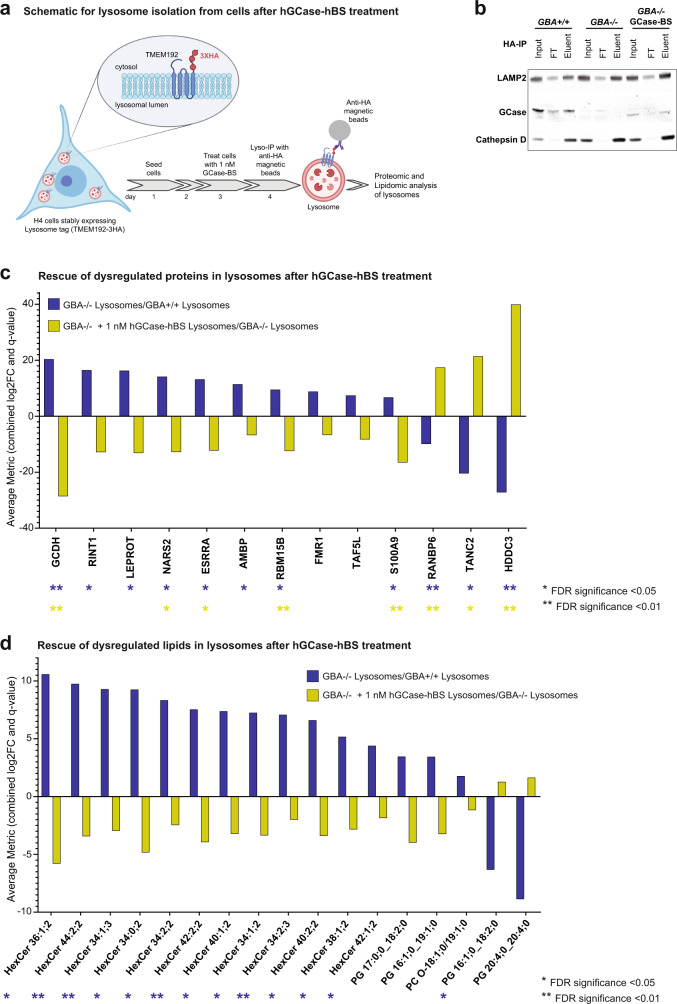
hGCase-hBS reverts lysosomal protein and lipid dysregulation in H4 *GBA-/-* cells. **a** Scheme for hGCase-hBS treatment and lysosome isolation from cells. H4 cells expressing lysosome-tag (TMEM192-3HA) were treated with 1 nM hGCase-hBS for 24 h, followed by cell lysis and lysosome isolation using anti-HA coated magnetic beads. Schematic created with BioRender.com. **b** Validation of lysosome enrichment after TMEM192-3HA-based Lyso-IP. Western blots showing enrichment of lysosomes after Lyso- IP as demonstrated by enrichment of bonafide lysosomal proteins LAMP2, Cathepsin D and GCase in eluent fraction compared to input. GCase is detectable in lysosomes 24 h after treatment of *GBA−/−* cells. Representative blots of 3 replicates. **c** Rescue of dysregulated lysosomal proteins upon hGCase-hBS treatment. Graph showing increased or decreased levels of lysosomal proteins in *GBA−/−* cells (blue bar) and its rescue upon hGCase-hBS treatment (yellow bar). **d** Rescue of dysregulated lysosomal lipids upon hGCase-hBS treatment. Graph showing increased or decreased levels of lysosomal lipid species in *GBA−/−* cells (blue bar) and its rescue upon hGCase-hBS treatment (yellow bar). Note: The lipid analysis performed does not report the number of carbons in the sphingoid base and the acyl chain (fatty acid chain) separately. Hence, the first number in the nomenclature used refers to the total number of carbons (sphingoid base + acyl chain). An Excel sheet consisting of each of the detected lipid species and its corresponding Swisslipids ID is provided in Supplementary Data File 3. FDR-corrected *p*-values are shown in (**c**) and (**d**) (**q-value <0.01; *q-value <0.05; *n* = 3 independent measurements), calculated for each contrast separately. Trends are also displayed (e.g. significance found in one contrast only), to highlight potential lipid/protein entities with opposite fold change in *GBA−/−* and rescue upon hGCase-hBS treatment. Source data are provided as a Source Data file. Schematics created with BioRender.com.

Having demonstrated the efficacy of the Brain Shuttle in delivering GCase to lysosomes in neuronal cell lines, we sought to investigate the consequences of reinstating lysosomal GCase on the molecular architecture of lysosomes. Since treatment of *GBA−/−* cells with 1 nM hGCase-hBS was sufficient to normalise GlcSph levels without saturating the system, we used this condition to better understand the lysosome-specific changes in proteins and lipids. To this end, we established an experimental paradigm to specifically enrich and profile lysosomes for proteomic and lipidomic perturbations upon hGCase-hBS treatment (Fig. 5a). We used a method to purify lysosomes from cells that relies on stable expression of the so-called Lysosome-tag, TMEM192-3XHA^37^. Expression of the Lysosome-tag facing the cytosolic side makes it possible to rapidly and efficiently immunoprecipitate lysosomes using anti-HA antibodies after cell lysis (Fig. 5a). Following treatment of cells with hGCase-hBS, we purified lysosomes in this way and confirmed they were intact by probing the presence of the lysosomal membrane protein, LAMP2 and soluble lysosomal lumen proteins, Cathepsin D and GCase (Fig. 5b, Eluent fraction). The isolated lysosomes, as well as whole-cell extracts, were subjected to global protein profiling using HRM^TM^ ID/ID + mass spectrometry. Proteomic analyses showing enrichment of bona fide lysosomal proteins within the isolated lysosomal fraction, as compared to whole-cell extracts, further validated the isolation protocol delivering intact lysosomes (Supplementary Fig. 5). The list for bona fide lysosomal proteins includes proteins shown to be of lysosomal origin by comparative proteomic analysis of lysosomes from mammalian cells^38,39^ (see also Supplementary Data File 1). Principal component analysis (PCA) of the proteomic dataset showed a clear separation between lysosomes and whole-cell extracts groups, as well as a clear separation between *GB*+/+ and *GBA−/−* groups (Supplementary Fig. 6). Several proteins were significantly upregulated or downregulated in both fractions from H4 *GBA−/−* cells as compared to those from H4 WT cells (Fig. 5c and Supplementary Fig. 7). Treatment of H4 *GBA−/−* cells with 1 nM hGCase-hBS was sufficient to readjust the altered protein levels both in the lysosomes (Fig. 5c) and whole-cell extracts (Supplementary Fig. 7a). One of the proteins that was significantly increased in GCase-deficient lysosomes is the pro-inflammatory mediator S100A9 (log2FC - *GBA-/-* lysosomes vs. *GBA*+/+ lysosomes: 1.77, Supplementary Data File 2). Interestingly, S100A9 has been shown to colocalise and co-aggregate with alpha-synuclein in Lewy bodies in PD patients and in vitro studies suggest that S100A9 might alter the aggregation kinetics of alpha-synuclein^40,41^. Treatment with hGCase-hBS could efficiently revert the observed increase in S100A9 levels in GCase-deficient lysosomes (log2FC - *GBA−/−* + 1 nM hGCase-hBS lysosomes vs. *GBA−/−* lysosomes: −2.49, Supplementary Data File 2). Another interesting protein that was significantly increased in GCase-deficient lysosomes and efficiently reverted by hGCase-hBS treatment is Estrogen related receptor alpha (ESRRA) (log2FC - *GBA−/−* lysosomes vs. *GB*+*/*+ lysosomes: 3.27; log2FC - *GBA−/−* + 1 nM hGCase-hBS lysosomes vs. *GBA−/−* lysosomes: −3.17). ESRRA has been shown to increase the cellular expression of monoamine oxidases (MOA) - a mitochondrial enzyme responsible for oxidation of dopamine. Moreover, parkin was shown to negatively regulate this process by ubiquitination and degradation of ESRRA^42^.

Using the same experimental paradigm employed in the proteomic study, we performed mass spectrometry-based shotgun lipidomic analysis of lysosomes and whole-cell extracts to assess the impact of hGCase-hBS on the lipid profile in GCase-deficient H4 cells. Many lipid species belonging to the hexosylceramide (HexCer) family are increased in lysosomes and whole-cell extracts lacking GCase and are efficiently reverted towards basal levels with 1 nM of hGCase-hBS (Fig. 5d and Supplementary Fig. 7b). Of note, our data suggests that beyond GlcSph, hGCase-hBS catalyses the breakdown of several species of HexCer within the lysosome that differ in chain length, double bonds, and hydroxylation. We also found that levels of lipid species belonging to the phosphatidylcholine (PC), phosphatidylglycerol (PG) and phosphatidylserine (PS) were significantly altered in GCase-deficient lysosomes and whole cells (Fig. 5d and Supplementary Fig. 7b). Notably, several of these altered lipid species were subsequently corrected to basal levels upon treatment with hGCase-hBS (Fig. 5d and Supplementary Fig. 7b). Taken together, our data suggests that GCase deficiency in cells leads to lipid changes beyond the known direct GCase substrates and that a subset of these altered lipid levels can be corrected in a rapid time frame after incubation with hGCase-hBS.

### GCase-BS proof of concept in vivo

**Fig. 6:**
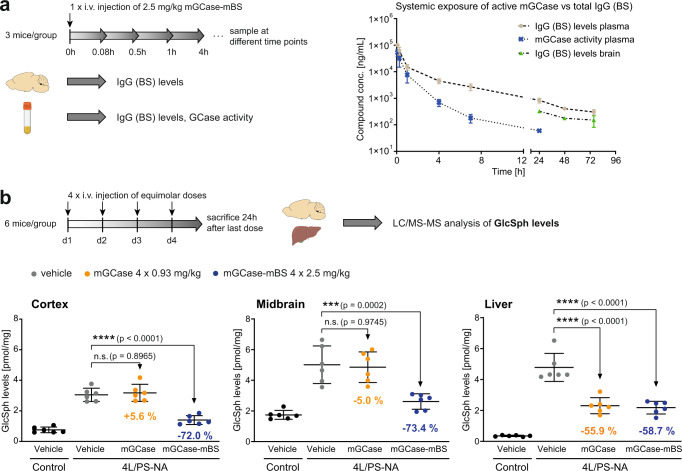
GCase-BS proof of concept in vivo. **a** PK study in *Gba*+*/+* mice to assess systemic exposure of mGCase-mBS in plasma and brain. *n* = 3 mice/group. **b** Multi-dose PD study in 4 L/PS-NA mice to compare equimolar doses of mGCase vs. mGCase-mBS. GlcSph levels were measured in cortex, midbrain and liver. *n* = 6 mice/group. Data are represented as group mean +/− SEM. Data was analysed by one-way ANOVA (Dunnett’s multiple comparisons test) comparing each treatment group to 4 L/PS-NA, vehicle. n.s. *p* > 0.05; ****p < 0.0001. Source data are provided as a Source Data file. Schematics created with BioRender.com.

We next performed a single-dose study using C57BL/6 mice in which mGCase-mBS levels were analysed measuring both the BS moiety (total IgG) of the construct by an IgG immuno-assay and GCase enzymatic activity using a chemical substrate. In this way we could assess the pharmacokinetics (PK) and stability of the molecule in vivo. The mGCase-mBS construct shows a high systemic clearance (>30 ml/h/kg). Initially, plasma levels measured with both assays were similar (5 min after injection), while for the entire period of observation, the exposure in terms of area under the curve (AUC) was approximately ¼ lower when enzymatic activity was measured as compared to the exposure measured using the IgG immune-assay (Fig. 6a). This suggests that the stability of the GCase domain was affected in the blood over time. As the uptake into the brain is the rate limiting step, assessing brain exposure by measuring total IgG levels from brain lysates showed a slightly lower but parallel pharmacokinetic profile (Cmax for total IgG in brain at 24 h: 2.1 nM). No active mGCase could be detected in plasma after 24 h, likely due to lack of sensitivity of the GCase enzymatic activity assay (LOQ = 0.4 nM).

To demonstrate proof of concept for the GCase-BS construct in vivo, initially we performed a multiple dose (4 doses of 2.5 mg/kg) study in a mouse model of GD which is named 4 L/PS-NA. These mice have a homozygous *Gba1* mutation (*Gba V394* *L/V394L*), a complete knock-out of *Psap* (PS-) substituted by a low-expressing mouse *Psap* transgene (NA)^43^. The mice exhibit neuronal phenotypes that are similar to those in GD2 or GD3 patients, *eg*. decreased GCase activity and a strong accumulation of GlcCer and GlcSph in the lysosomal compartment^43,44^. Assuming that the pharmacokinetics in the brain in these mice also run in parallel to the blood profile, the rationale for the dosing regime was to generate high enough brain exposures to allow target engagement and so induce a clear pharmacodynamic effect on relevant markers. We found that at baseline, compared to control littermates, the 4L/PS-NA mice showed an elevation of GlcSph levels to 4-fold (cortex), 3-fold (midbrain) or even 13-fold (liver). 24 h after the last dose of mGCase-mBS in the 4 L/PS-NA mice, lipid levels in both the cortex and midbrain were reduced by ~72% (Fig. 6b). Non-conjugated mGCase alone was not effective at reducing lipids in multiple brain regions suggesting that the BS module is key for reducing brain lysosomal GlcSph (Fig. 6b). In the liver, however, both mGCase and mGCase-mBS resulted in ~60% normalisation of substrates suggesting they are similarly potent in the liver which was unexpected based on the observation that TfR is more efficient (Fig. 5b). This unexpected observation might be explained by differential expression patterns of M6PR and TfR in this tissue compared to brain tissue including almost no TfR in hepatocytes (Supplementary Fig. 9c). Similar results were obtained in mice carrying only the *Gba1* mutation (*Gba V394L/V394L*) alone (Supplementary Fig. 8a). In summary, these results suggest that the Brain Shuttle module not only increases lipid reduction potential but also promotes crossing of the BBB into the brain parenchyma in two *Gba1* murine models.

**Fig. 7:**
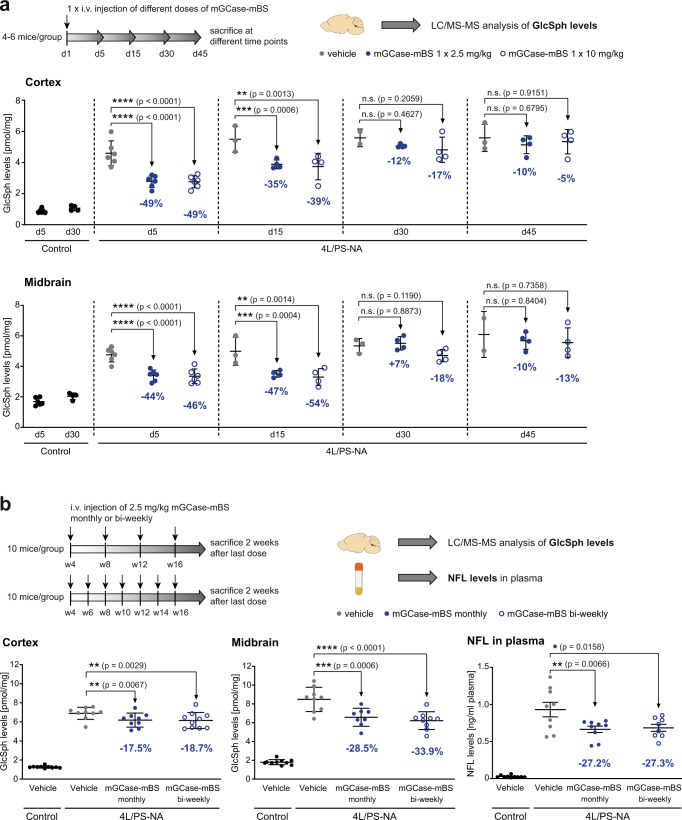
GCase-BS longitudinal effects in vivo. **a** Single-dose PD study in 4 L/PS-NA mice to inform about duration of GlcSph lowering in cortex and midbrain. GlcSph levels rebounce between 15–30 days post administration. *n* = 4–6 mice/group. **b** Chronic study in 4 L/PS-NA mice with monthly or bi-weekly dosing frequency. GlcSph levels in cortex and midbrain as efficiency readout. NFL levels in plasma as readout for neurodegeneration. *n* = 10 mice/group. Data are represented as group means +/− SEM. Data was analysed by one-way ANOVA (Dunnett’s multiple comparisons test) comparing each treatment group to 4 L/PS-NA, vehicle. n.s. *p* > 0.05; *p < 0.05; **p < 0.01; ***p < 0.001; ****p < 0.0001. Source data are provided as a Source Data file. Schematics created with BioRender.com.

Subsequent single dose dose-response experiments were performed at doses ranging from 0.2 mg/kg to 2.5 mg/kg and results suggested that doses below 2.5 mg/kg are not sufficient to significantly lower brain lysosomal lipid levels (Supplementary Fig. 8b). To gain more insight into suitable dosing frequencies and the trajectories of pathological lipid rebound after dosing, we injected a single-dose of either 2.5 mg/kg or 10 mg/kg and analysed tissue at various time points after dosing in 4 L/PS-NA mice (4–6 mice per group). GlcSph analysis revealed that both doses similarly led to ~50% reduction of substrate as benchmarked to control animals at day 5 post injection. The kinetics of changes of GlcSph levels revealed that at 15 days post injection, GlcSph levels were still significantly lower in both cortex and midbrain and that it took up to 45 days to return to levels of GlcSph seen in untreated animals. These results suggest that lipid reduction after treatment is sustained and a bi-weekly or monthly dosing frequency could be sufficient to reach beneficial effects (Fig. 7a).

To test whether this was correct, we performed a multi-dose chronic study, where we injected 4 L/PS-NA mice monthly or bi-weekly with 2.5 mg/kg of mGCase-mBS starting from 1 month of age for 3 months. In this study, we monitored GlcSph levels in the brain and, as a biomarker of neurodegeneration, NFL levels in plasma biomarker of neurodegeneration previously reported to be elevated in this model^44,45^. Two weeks after the last injection, in both cortex and midbrain, there was an ~18% and ~30% reduction of substrate levels in cortex and midbrain, respectively (Fig. 7b). We did not observe a marked difference between the bi-weekly and monthly dosing regimens. Notably, NFL levels were 35-fold elevated in the 4LPS-NA mice plasma compared to controls. Using both dosing regimens we observed significant reductions to almost 30% of untreated 4LPS-NA. These results suggest that both bi-weekly or monthly dosing is adequate to lower lipids sufficiently to alter the trajectory of neurodegeneration in an aggressive GD model (Fig. 7b).

## Discussion

The classical view of the lysosome has recently been expanded by the discovery of new roles of lysosomes in nutrient sensing, transcriptional regulation, and metabolic homeostasis^46^. At the subcellular level, LSDs manifest in abnormal intra-lysosomal accumulation of metabolites due to defects in one or multiple catabolic pathways caused by genetic defects that lead to reduced levels of lysosomal enzymes^47^. The clinical manifestations of LSDs vary widely but neurological symptoms are common features^48^. Restoring the levels of the missing enzyme is a highly effective treatment and is standard of care in many different types of LSDs. However, the recombinant enzymes used for ERT lack the ability to cross the BBB. This leads to no or poor brain exposure of therapeutic enzymes and subsequent failure in reversing neurological complications in patients.

There is a compelling body of preclinical and clinical data suggesting that increasing GCase activity in the brain can revert the underlying lysosomal dysfunction, thus having a marked impact on disease trajectory^49,50^. Current ERT for GCase, which is an effective treatment for GD1^51,52^, has limitations which we could address to enhance targeting of CNS lysosomal dysfunction. The GCase-BS construct not only passes the BBB endothelium, but it is also more efficiently reducing accumulated GlcSph in multiple relevant human cellular systems (Fig. 3 and Supplementary Fig. 3). GlcSph is formed within lysosomes as a result of the accumulation of GlcCer and acid ceramidase activity^5^. It is therefore conceivable that the observed reductions in GlcSph levels could result from the reduction of accumulated GlcCer that acts as source for GlcSph formation or from direct hydrolysis of GlcSph by GCase-BS. While one or the other, or indeed both, are formal possibilities, we show in any event that GCase-BS can reach and then act within the lysosome of our GD cellular model to reduce GlcSph levels and multiple GlcCer species (Fig. 2 and Fig. 3). Collectively, our data suggests that fusion proteins comprising GCase and TfR binders represent a promising therapeutic approach for GD type 2 and 3 and potentially other *GBA1*-associated neurological diseases.

Recently it has been shown both preclinically and in human clinical trials that the lysosomal enzyme iduronate 2-sulfatase (IDS) coupled to TfR binders are able to pass the BBB by demonstration of CNS target engagement (*eg*. CSF/NFL)^53,54^. This effort has culminated in the approval of JR-141 (IDS TfR binder) for mucopolysaccharidosis (MPS) type I for central disease manifestations in Japan^55^. Recent work has also highlighted that this approach extends to other lysosomal proteins including progranulin. Since the concept of TfR binding to pass the BBB to treat MPS2 and neuronal ceroid lipofuscinosis (NCL) has been validated in humans^56,57^, we now extend these findings for neuronopathic GD.

ERT for GD type 1 is based on targeting macrophages by terminal mannose groups in its glycans which bind to the MR^30,31,58^. Since the MR is not expressed in neuronal cells a different mechanism is required. Most of the newly synthesised lysosomal enzymes get phosphorylated at their mannose residues to be sorted to early and late endosomes^59^. Since the M6PR-CI is not only localised at the trans-Golgi network and endosomes but also at the plasma membrane, exogenously delivered GCase could be retrieved by M6PR-CI and sorted to the endolysosomal system, and subsequently to the lysosome^36,59,60^. This mechanism might represent the route taken in the CNS for ERTs including GCase (Fig. 4e). In the case of GCase-BS, we demonstrated that uptake, lysosomal exposure and reduction of pathological GlcSph (and GlcCer) are far more efficient and potent than the enzyme on its own. We show that this “mechanism of action” is predominantly dependent on the TfR and not the M6PR. Interestingly, the GCase construct with an inactive TfR binder (hGCase-NB) displays hardly any uptake or substrate reduction indicating that this construct is unable to effectively use the M6P pathway through M6PR-CI sorting (Fig. 4). This suggests that the size of the non-TfR binding antibody interferes with efficient uptake. However, when an active TfR binder is used as in the GCase-BS construct, strong cellular uptake and robust substrate reduction is seen. Thus, in the neuronal cells used in this study, the TfR provides an efficient pathway into lysosomes. Importantly, at the BBB, this TfR sorting pathway in polarised endothelial cells is different. Here, a portion of GCase-BS is transported across the endothelium into the brain parenchyma if the engagement with the TfR possesses certain features, including a monovalent binding-mode, which appears to prevent lysosomal sorting within the endothelial cells at the BBB^21^. Taken together, data presented in this work shows that the TfR pathway can provide both productive transport across cells at the BBB and subsequently enhance uptake and efficacy in hydrolysis of lysosomal glycolipids in neuronal cells within the brain. We furthermore showed that in patient-derived macrophages and microglia, where the MR is highly expressed, the efficacy of hGCase-hBS is higher when compared to imiglucerase suggesting that the TfR lysosomal sorting is conserved across different cell types (Supplementary Fig. 3). Hence, the Brain Shuttle module could even enhance efficacy in peripheral cell types, which might be relevant for other LSDs where peripheral manifestations are not well managed with conventional ERT (*eg*. targeting skeletal muscle^61^). It is also important to appreciate that the cargo associated with the BS in a fusion construct may influence trafficking of the construct. For example, if the cargo is a high-affinity antibody against a specific target in the brain, it is likely that the BS construct will be directed towards this target due to the stronger binding compared to the binding to the TfR. Therefore, whether other lysosomal enzymes coupled to TfR binders share features seen here for GCase-BS, including increased uptake and efficacy, remains to be determined.

Upon systemic administration of mGCase-mBS rapid clearance of the construct from blood is observed (Fig. 6a). Notably, in blood, GCase enzymatic activity seems to decline much faster than the shuttle domain of the construct. Only about 0.1% of the injected enzymatic activity is detectable in circulation after 24 h. This suggests that the GCase enzyme is inactivated at neutral pH and is likely driving the clearance of the construct from the peripheral compartment. This observation highlights current limitations that could be further optimised, including reducing clearance and increasing enzyme stability. The short half-life and stability of the GCase-BS will ultimately limit the amount available for brain uptake and efficacy. There are reports that GCase mutants may have increased stability suggesting the possibility for further enzyme engineering^62,63^. Alternatively, GCase-BS could potentially be combined with pharmaceutical chaperones to increase stability in the blood. In the context of Pompe disease, recombinant alglucosidase alfa in combination with the pharmacological chaperone miglustat, stabilises the enzyme, improves its pharmacokinetic properties and leads to better function compared to enzyme alone^64,65^. This strategy could be applied to GCase-BS using a known GCase chaperone such as ambroxol or isofagomine^66^.

Mouse models of *Gba1*-associated PD have limitations that impact their utility for the purpose of studying disease biology. Such models, for example, have very little accumulation of lipids in the brain^67^. We therefore leveraged the 4LPS/NA model that harbours a homozygous knock-in of *Gba V394L/V394L*, a complete knock-out of *Psap* and a low-expressing mouse *Psap* transgene. This mouse model shows extensive accumulation of GlcSph in the brain and was ideal for the purpose of monitoring pharmacodynamic effects of the GCase-BS fusion protein. The decreased level of *Psap* leads to deficiency in saposin C, a co-activator of GCase, and certainly affects GCase efficacy and other lysosomal functions whereby the effects seen in vivo might be underestimated^68,69^. Nonetheless, we showed that a single dose of mGCase-mBS reduced GlcSph levels by nearly 50% relative to WT levels, while four daily doses resulted in ~70% reduction in relation to WT levels (Fig. 6b). These results imply mechanistically that while the brain in vivo effect is in part driven by the maximum concentration (Cmax), supported by the in vitro work showing that lysosomal hydrolysis is fast, the capacity of the TfR at the BBB to shuttle mGCase-mBS can become saturated. This view is supported by observations showing that doses from 1–10 mg/kg result in a limited dose-response relationship (Fig. 7a).

Within the liver, both the GCase enzyme and the mGCase-mBS construct are highly active, verifying that the GCase ERT is functional and active in vivo outside the brain yet cannot penetrate into the CNS. Similar efficacies of both mGCase and mGCase-mBS in liver could be explained by different expression levels of M6PR and TfR in this tissue compared to brain cells (Supplementary Fig. 9c). Another notable observation is the marked and sustained reduction in GlcSph levels in the brain after ending treatment. Both in cortex and midbrain, a significant reduction of GlcSph is observed up to 15 days post treatment and clear trends even up to 45 days were seen (Fig. 7a). Recently it has been shown that the NFL levels in the CSF and plasma in this 4 L/PS-NA GD mouse model are 9-fold higher in plasma and 70-fold elevated in the CSF compared to wild-type controls^45^. Similar increases in plasma NFL was observed in this study (Fig. 7b). 12-weeks treatment with bi-weekly/monthly dosing of the mGCase-mBS, was able to significantly reduce these abnormal NFL levels. Since NFL is found in large myelinated axons of neurons, this measurable therapeutic effect likely originates within neurons in the CNS. The reduction of NFL is striking since the model has a component of neurodegeneration that is independent of GCase, driven by the deficiency of *Psap*.

CSF GlcSph is a putative translational biomarker for GD that is downstream to elevating GCase in brain. However, as highlighted by the recent venglustat clinical trial, reduction of key pathological lipids will not be sufficient to determine whether *GBA1*-dependent lysosomal homeostasis has been restored^70,71^. This is why we monitored protein and lipid changes in both whole cell lysates and purified lysosomes from a GD cellular model. We identified both proteins and lipids that were abnormally regulated in GD cells and rapidly reverted after addition of hGCase-hBS (Fig. 5 and Supplementary Fig. 7). Future work could address the role of these molecular changes on *GBA1*-associated neurodegeneration and ultimately may serve as proximal *GBA1* pathway lysosomal biomarkers downstream of target engagement. A key translational biomarker may require rapid lysosomal purification of peripheral cells to demonstrate functionality in the lysosomal compartment^46^.

In summary this work suggests that GCase-TfR binder fusion proteins can correct lysosomal deficiencies and hence represent an attractive therapeutic avenue for GD and potentially also for *GBA1*-associated neurodegeneration. We furthermore propose opportunities for future optimisation of such constructs and provide the foundation for potential biomarkers that can be employed to examine efficacy of lysosome-targeted therapeutics in a translational context.

## Methods

### Reagents and antibodies

Chemicals were purchased from Sigma Aldrich, if not stated otherwise.

Antibodies used to perform immuno-based experiments were: rb mAb to hGCase (clone EPR5143(3); Abcam, #ab128879), ms mAb to hGCase (clone 1/17; in-house production), rb mAb to LAMP1 (clone D2D11; Cell Signaling Technology, #9091), rb mAb to TFR (Abcam, #ab214039), rb mAb to M6PR cation-independent (clone EPR20584; Abcam, #ab124767), rb mAb to M6PR cation-dependent (clone EPR7691; Abcam, #ab134153), ms mAb to LAMP2 (clone H4B4; Thermo Fisher. #MA1-205), rb mAb to Cathepsin D (clone EPR3057Y; Abcam, #ab75852), mAb anti-hFab(kappa) (in-house production), HRP-conjugated pAb anti-hFab(CH1) (Creative Biolabs, #MOB-0361MC), PE-conjugated gt anti-hIgG (Fc-specific) (Jackson ImmunoResearch, #109-116-170), HRP-conjugated rb pAb to GAPDH (Abcam, #ab9385). Antibody dilution is indicated in respective methods sections.

### Cell cultures

#### Human neuroblastoma cells (H4)

H4 cells were maintained in DMEM/F-12 (#11039-021) supplemented with 10% fetal bovine serum (#A31605-01) and penicillin (100 U/ml), streptomycin (100 µg/ml) (ThermoFisher).

#### Human neurons

Neural stem cells (NSCs) were generated and differentiated into neural cultures as described previously^72^. For patterning, NSCs were plated on polyornithin-/laminin-coated culture flasks at 1E4 cells/cm² in DMEM/F-12 with GlutaMax (#31331093) and neurobasal medium (#21103049) supplemented with 1X B27 (ThermoFisher #12587010), 1X N2 (ThermoFisher #17502048), 50 µM 2-mercaptoethanol (ThermoFisher #31350010) and 100 ng/ml FGF-8 (Peprotech), 200 ng/ml sonic hedgehog (Peprotech), and 100 μM ascorbic acid 2-phosphate (Sigma) and cultured for one week. For differentiation, the resultant progenitors were plated at 5E4 cells/cm² in basal medium supplemented with 20 ng/ml BDNF, 10 ng/ml glial cell-derived neurotrophic factor (GDNF; Peprotech), 500 μM dibutyryl cyclic AMP (Sigma), and 100 μM ascorbic acid 2-phosphate.

##### Cell line source

Human Embryonic Stem Cell Line SA001, Cellartis AB; NIH Human Embryonic Stem Cell Registry no. 0085; origin info: male, ethnicity and age N/A.

#### Mouse immortalised primary neurons

Mouse immortalised primary neurons were generated and characterised previously^26^. The cells were maintained in neurobasal medium supplemented with 1X B27, 1X GlutaMax (ThermoFisher #35050061), penicillin (100 U/ml), streptomycin (100 µg/ml), laminin (1/500).

#### Human iPSC-derived cells

The human iPSC lines used for macrophage and microglia differentiation in this study included a healthy control case (BIONi010-C, K3P53; male; age 15–19; available from https://hpscreg.eu/cell-line/BIONi010-C) and a PD patient bearing the heterozygous *GBA N370S* variant (STBCi025-C, SFC834-03-10; rs76763715: p.Asn409Ser, male; age 72; available from https://hpscreg.eu/cell-line/STBCi025-C).

Both lines were generated at the StemBANCC consortium and deposited at the European Bank for induced pluripotent Stem Cells (EBiSC). The lines are fully consented for research use (Berkshire Research Ethics Committee, Approval number 10/H0505/7) and quality controlled by EBiSC.

The lines were tested for sterility, absence of mycoplasma contamination and for the absence of unwanted genetic abnormalities by karyotyping and tested for common copy-number variations. The identity of the lines was confirmed by Short Tandem Repeat (STR) analysis and pluripotency was confirmed by flow cytometry.

All cells were kept at 37 °C in a humidified 5% CO_2_ atmosphere.

### Animals

#### C57BL/6 mice

Pharmacokinetics of mGCase-mBS were assessed in-house using male C57BL/6 mice. Blood samples during the course of experiment were taken from the sublingual vein under isoflurane anaesthesia. For terminal blood and tissue collection, animals were terminally anaesthetized with isoflurane and decapitated. Terminal blood samples were taken by cardiac puncture. The study was approved by the Cantonal Veterinary Office (CVO) Basel-Stadt.

#### Gba1 D409V/D409V mice

Heterozygous *Gba D409V/*+ breeders were purchased from Jackson Laboratory (019106; www.jax.org/strain/019106). A local breeding colony was established and maintained by mating heterozygous mice at Simon Fraser University. The genotype of mice was confirmed using a genomic DNA sample isolated from an ear snip by standard PCR to analyse the mutant gene product (387 bp) and WT gene product (279 bp). The forward primer sequence was CAG TTC ACA CAG TGT TGG AGC and the reverse primer sequence was AGG TGA TCG TAT TTC AGT GGC. Additional genotyping information and protocols were followed as provided in “Technical Support” on The Jackson Laboratory *Gba1 D409V* mouse model webpage (www.jax.org/strain/019106). Protocols governing the use of animals were approved by the Animal Care Review Committee of Simon Fraser University and were in compliance with guidelines published by the Canadian Council of Animal Care (CCAC).

#### 4 L/PS-NA mice

For in vivo studies, we used the 4 L/PS-NA mouse model resembling some neuronopathic phenotypes of GD2 and 3. These animals harbour a homozygous point mutation in the *Gba1* locus (*Gba V394L/V394L*), a complete knock-out of *Psap* (PS-) and a low-expressing mouse *Psap* transgene(NA)^43^. Control littermates (WT for *Psap*) were used as a baseline.

Additionally, we used a mouse model that only harbours the homozygous V394L mutation in the *Gba1* locus (*Gba V394L/V394L*) and compared results to C57BL/6 mice (no littermates).

Breeding and studies were carried out at QPS Austria GmbH according to respective animal handling regulations. The QPS Austria animal facility is fully accredited by the Association for Assessment and Accreditation of Laboratory Animal Care (AAALAC). All studies carried out were approved by the Office of Styrian Government, Department 13, Environment and Regional Planning (Licenses ABT13-21043/2019 and ABT13-157513/2020) and complied with the Austrian Animal Welfare Council (AAWC).

Mice were allocated to groups in a randomized manner. Randomization of group allocation was done per cage. Animals were assigned to different starting groups (cohorts) comprising animals of all treatment groups if possible. Cohorts of mixed sex animals were used. The mice were 4–8 weeks old at the start of the studies.

After the last treatment, animals were terminally anaesthetized by intraperitoneal injection of pentobarbital (600 mg/kg) and samples were collected immediately.

All animals were housed in individual ventilated cages on standardized rodent bedding supplied by Rettenmaier. Each cage contains a maximum of five mice. The room temperature was maintained at 20 °C to 24 °C and the relative humidity is maintained at 30% to 70%. Animals were housed under a constant light-cycle (12 h light/dark). Dried, pelleted standard rodent chow (Altromin) as well as normal tap water is available to the animals ad libitum.

### Isolation and culture of embryonic cortical neurons from *Gba D409V/D409V* mice

WT (*Gba*+*/+*) and homozygous (*Gba D409V/D409V*) mice were selected from the colony and timely breeding was carried out by mating of WT x WT and homozygous x homozygous. At E16.5 individual cortices were dissected out from individual embryos, pooled together for each genotype, and collected in 96 well plates. Cortices were then washed with 10 ml of dissecting buffer (HBSS supplemented with Pen/Strep). For each wash, 10 ml of fresh dissecting buffer was added and tissues were allowed to precipitate down to the bottom of each falcon tube. Buffer was then carefully decanted and this washing process was repeated five times. Cortex tissues were then incubated with 1 X Trypsin EDTA at 37 °C for 20 min. After that, the supernatant was carefully decanted and 10 ml of fresh dissecting buffer was added. Once tissues had settled to the bottom of the tune the buffer was carefully decanted and this process was again repeated five times. Next, 1 ml of plating medium prepared using BrainPhys™ Primary Neuron Kit (STEMCELL Tech, Cat. #05794) was added and cells were dissociated gently, but thoroughly, by pipetting up and down for 30-60 times using a pipette equipped with a 1 ml tip. Cells were next passed through a cell strainer (40 μM), rinsed with 1 ml of plating medium and cells were twice rinsed with 1 ml of plating medium. Cells were then counted and the cell concentration was adjusted to be consistent among samples using plating medium. Cells of each genotype were then seeded on high-content imaging 96-well microplates (Corning 4680) precoated with poly-D-Lysine (Gibco, Cat. # A3890401) at a density of 7.5E4 cells/cm^2^ with a final volume of 100 μl/well. Starting from day 5 after seeding, half of the medium was changed every three days with Complete BrainPhys^TM^ Neuronal Medium prepared using a BrainPhys™ Primary Neuron Kit (STEMCELL Tech, Cat. #05794).

### Immunolabeling and mGCase-mBS assays in primary mouse neurons

On day 11 after seeding, primary mouse neurons in designated wells were treated with the specific GCase inhibitor AT3375 (10 μM) to serve as negative controls. On day 12 after seeding, neurons were incubated with either mGCase or mGCase-mBS at different concentrations for 2 h. Neurons were then incubated with 10 μM of LysoFQ-GBA for 30 min. Processing of LysoFQ-GBA by GCase within cells was terminated by the addition of 10 μM of AT3375. After staining nuclei with Hoechst 33342, live neurons were imaged using a high-content imaging microscope (Molecular Devices ImageXpress). Acquired images were analysed using MetaXpress (Molecular Devices) to calculate integrated fluorescence intensity arising from LysoFQ-GBA and count the number of cells in each well based on counting of nuclei. Lysosomal GCase activity was represented as integrated fluorescence intensity of LysoFQ-GBA product / cell. Immediately after the GCase live-cell assays, neurons were fixed using 4% PFA at RT for 20 min and then washed thrice with PBS. After blocking with 5% BSA in PBS for 1 h, neurons were incubated overnight at 4 °C with primary antibody against beta-tubulin II (TUJI). The next day, primary antibodies were removed, and the cells were washed 5 times with PBS times and then incubated with fluorescent secondary antibody. After washing 5 times with PBS, antibody-labelled cells were imaged by widefield microscopy (ImageXpress).

### Generation of H4 KO cell lines

CRISPR/Cas9 gene editing was performed to generate several KO H4 cell lines: In brief, H4 GBA KO cells were seeded at 1E5 cells/well into a 6 well-plate and transfected with RNPs at a 1.8:1 ratio (sgRNA:Cas9 nuclease) by lipofection. Lipofection reagents were purchased from Thermo Fisher Scientific (Lipofectamine™ CRISPRMAX™ Cas9 Transfection Reagent #CMAX00015). Media was changed to full growth media after 18 h and cells were subjected to limiting dilution to obtain monoclonal cell populations.

Synthetic sgRNAs were purchased from Synthego:

1. TFRC: guide #1: G*U*G*AUCGUCUUUUUCUUGAU guide #2: A*A*A*UGCUGACAAUAACACAA guide #3: A*G*A*UGGCGAUAACAGUCAUG
2. M6PR-CD: guide #1: A*A*U*CAACAAAAGUAAUGGGA guide #2: U*U*C*AGGGUGUGCCGGGAAGC guide #3: U*A*C*AGCUUUGAGAGCACUGU
3. M6PR-CI: guide #1: U*U*G*AAUUGUGCAGGUAACGA guide #2: C*G*U*GUCCCAUGUGAAGAAGU guide #3: C*G*U*CGGUGGCACCGCAGAGG

Absence of respective proteins was determined by Western Blot analysis. In brief, 1E6 cells were lysed in RIPA buffer, supplemented with protease inhibitors (cOmplete; Roche, #11873580001), and 10 µg of total protein were subjected to SDS-PAGE (Invitrogen NuPAGE system). Semi-dry transfer at 23 V for 6 min was used to transfer proteins onto a nitrocellulose membrane, which was blocked in 5% (w/v) non-fat dry milk/Tris-buffered saline + 0.05% (v/v) Tween20 and subsequently incubated with primary antibody at 1/1000 o/n at 4 °C. After 3 washes in TBST, HRP-labelled secondary antibody was incubated for 1.5 h at RT at 1/10000 in 1% milk/TBST. Proteins were detected using the SuperSignal West Dura Extended Duration Substrate Kit (ThermoFisher). Images were taken using the ChemiDoc MP Imaging System (BioRad).

### Expression and purification of recombinant human and mouse GCase-BS

The recombinant hGCase and mGCase as well as hGCase-hBS and hGCase-NB were expressed in Schneider S2 cells (*Drosophila* cell line), using the expression vector pExpreS2_1-A. hGCase and mGCase constructs were designed to express with a C-terminal His_8_-tag including a glycine-serine (GS) linker and a sortase recognition site (Sor). hGCase-hBS was designed by fusing one chain of a human IgG1 Fc portion devoid of Fcγ receptor binding to the C-terminus of human GCase and the other chain N-terminally to an anti-human TfR binding Fab using knob-into-hole technology. mGCase-mBS was expressed in two parts (mGCase-Sor and anti-mouse TfR binding Fab) and subsequently coupled by sortase-mediated site specific conjugation.

To purify mGCase or hGCase, cell supernatant was filtered and passed through a HiTrap Con A 4B column (GE Healthcare) using HiTrap Con A-Buffer (20 mM Tris/HCl at pH 7.4 with 0.5 M NaCl, 1 mM MnCl_2_, 1 mM CaCl_2_, 0.02% (v/v) NaN_3_ and 0.5 M Methyl α-D-mannopyranoside for elution). Eluted target protein was further purified with a HisTrap HP column (GE Healthcare) using HisTrap-Buffer (50 mM HEPES at pH 7.6 with 0.5 M NaCl, 0.02% NaN_3_ and 0.5 M imidazole for elution) followed by a hydrophobic interaction chromatography (HIC) with a Toyopearl Butyl-M 650 HIC column (Tosoh Bioscience) using HIC-Buffer (20 mM MES at pH 5.5 with 0.5 M KCl, 0.02% NaN_3_) for binding and 80% (v/v) ethylene glycol for elution.

To purify hGCase-hBS or hGCase-NB, cell supernatant was cleared by filtration and loaded onto a HiTrap Con A 4B column (GE Healthcare) as the first purification step. HiTrap Con A-Buffer (20 mM Tris/HCl at pH 7.4 with 0.5 M NaCl, 1 mM MnCl_2_, 1 mM CaCl_2_, 0.02% (v/v) NaN_3_ and 0.5 M Methyl α-D-mannopyranoside for elution) was used. Eluted target protein was further purified via a Capture Select KappaXL column (GE Healthcare) column using 25 mM Tris/HCl at pH 7.0, 25 mM NaCl, 5% (v/v) glycerol, 0.02% NaN_3_ as binding buffer. An additional wash step with binding buffer and 1% (w/v) CHAPS was included to remove endotoxins. The target protein was eluted with 20 mM citric acid at pH 3.5, 0.1 M glycine, 5% (v/v) glycerol and 0.02% NaN_3_ from the column. The pH was adjusted directly after protein elution to pH 6.0.

All purified constructs were finally dialysed against a slightly acidic solution (20 mM histidine, 140 mM NaCl, pH 6.0) for further experiments.

### Biochemical characterisation of GCase-BS

The enzymatic activity of the various GCase(-BS) constructs was determined using the fluorogenic substrate resorufin-β-D-glucopyranoside (res-β-glc; Sigma-Aldrich). GCase cleaves this substrate to glucose and resorufin. The product formation of resorufin was measured over time with excitation at a wavelength of λ = 535 nm and emission at λ = 595 nm in the assay buffer containing 50 mM citric acid pH 6.0, 50 mM KP_i_, 110 mM KCl, 10 mM NaCl, 1 mM MgCl_2_ and 1% DMSO at 37 °C. Prior to the kinetic measurements, all assay components were pre-warmed to assay temperature and the fluorogenic substrate was kept in the dark. The reaction was started by adding GCase to a final concentration of 25 nM. Raw data from the fluorescence plate reader (Spectramax i3, Molecular Devices; Software: SoftMax Pro 7) were aggregated in Microsoft Excel and analysed using GraphPad Prism 8.4.2.

To assess both GCase enzymatic activity and levels of IgG of the GCase-BS molecules after incubation in 10% mouse plasma, a capture assay was performed using a mAb anti-hFab(kappa) (in-house production) as capture antibody. After subsequent washes, GCase enzymatic activity was determined using Amplex™ Red Glucose/Glucose Oxidase Assay Kit (Thermo, #A22189) and, from the same sample, IgG levels were measured using an HRP-conjugated pAb anti-hFab(CH1) (Creative Biolabs, #MOB-0361MC). Data acquisition was done using a multimode plate reader (SpectraMax Paradigm, Molecular Devices; Software: SoftMax Pro 7).

### FACS-based assessment of TfR binding

The binding of the GCase-BS fusions was tested using mouse-TfR expressing cell line BA/F3 (DSMZ, ACC-300) or human-TfR expressing CHO cells (ATCC, CCL-61, transfected to stably overexpress human TfR). Briefly, suspension cells were harvested, counted, checked for viability and re-suspended at 2 million cells per ml in FACS buffer (PBS with 0.1% BSA). 100 µl of the cell suspension (containing 0.2 million cells) were incubated in round-bottom 96-well plates for 1 h at 4 °C with increasing concentrations of the GCase fusions (10 pM to 1 µM). Cells were then washed twice with cold PBS/5%FBS, re-incubated for further 30 min at 4 °C in the dark with a labeled secondary antibody (PE-conjugated, goat-anti-hu IgG (Fc-spec.)) from Jackson ImmunoResearch #109-116-170 at a dilution of 1:100, and washed twice with cold PBS/5% FBS. Fluorescence was analysed by FACS using a BD FACSCanto™ II (Software FACS Diva and FlowJo 10.6.2). Binding curves and EC_50_ values were obtained using GraphPad Prism 7.

### Cellular assays to assess uptake, lysosomal activity, potency and subcellular localisation of GCase-BS

GCase-deficient cell lines were used to determine uptake, lysosomal activity and potency of GCase-BS molecules.

To assess cellular uptake, the activity of GCase was determined from whole cell lysate. Cells were seeded at 5E4 cells/well into a 96-well plate and maintained at 37 °C, 5% CO_2_, 85% humidity for 16–18 h. Cells were then treated with a range of concentrations of the various GCase-BS molecules for 2 h. Subsequently, cells were washed once with PBS and lysed in 30 µl lysis buffer (0.05 M citric acid, 0.05 M KH_2_PO_4_, 0.05 M K_2_HPO_4_, 0.11 M KCl, 0.01 M NaCl, 0.001 M MgCl_2_, pH 6.0 with 0.1% (v/v) TritonX-100, supplemented with freshly added protease inhibitor). 10 µl of cell lysate were mixed with 10 µl of 10 mM resorufin-β-glucopyranoside and baseline fluorescence was measured at t_0_ immediately. The build-up of fluorescent product (resorufin) was measured after incubation for 2 h at 37 °C (*λ*_*ex*_ = 535 nm and *λ*_*em*_ = 595 nm) indicating GCase activity. Data was acquired at a multimode plate reader (SpectraMax Paradigm, Molecular Devices; Software: SoftMax Pro 7). Data was normalised to WT cells.

To assess lysosomal activity, a fluorescence-quenched GCase substrate (LysoFQ-GBA) was used that only emits light when GCase is hydrolysing it (and therefore releasing the quencher). Cells were seeded at 1E4 cells/well into a 96-well plate and maintained at 37 °C, 5% CO_2_, 85% humidity for 16–18 h. Cells were then treated with a range of concentrations of the various GCase-BS molecules for 2 h. Subsequently, cells were washed with PBS and a mix of FQ-7 and SiR lyso kit were added for 1 h at 10 µM and 5 µM respectively. Cells were washed once more with PBS and Hoechst (2 µM final) was added for 3 min. Cells were imaged live using the Opera Phenix Plus (Perkin Elmer; Software: Harmony 5.1) with a 40X water objective.

To assess potency of the GCase-BS molecules, cells were seeded at 2E4 cells/well into a 96-well plate and maintained at 37 °C, 5% CO_2_, 85% humidity for 16–18 h. Cells were then treated with a range of concentrations of the various GCase-BS molecules for 48 h. Cells were washed once with PBS and dry cell layer was deep frozen at −80 °C. Cells were thawed and subsequently lysed by adding distilled water and methanol containing internal analyte standards. Samples were evaporated to dryness, reconstituted in acetonitrile/water 90/10 (v/v) with 1% DMSO and analysed by LC-MS/MS. Lipids were simply quantified using the peak area ratio analyte/internal standard (=response). Data acquisition and analysis was done using MassLynx V4.1 SCN950 (Copyright 2015 Waters Inc.).

To assess cellular localisation of the various constructs, immunocytochemistry labelling was performed in H4 cells. Cells were seeded at 5E3 cells/well into an imaging-compatible 96-well plate and treated with the molecules for 2 h. Subsequently, cells were washed once with PBS and fixed using 4% PFA for 10 min at RT. After fixation, cells were washed 3 times with PBS and blocked with 1% donkey serum, 1 mg/ml saponin, 0.75 mg/ml glycine in PBS for 2 h at RT. Primary antibody was incubated o/n at 4 °C in Ab dilution buffer (0.1% BSA, 1 mg/ml saponin in PBS). After 3 washes in PBS, secondary antibody was incubated in Ab dilution buffer for 2 h at RT. Cells were counterstained with DAPI to label the nuclei and imaged using a 40X water objective at Opera Phenix Plus (Perkin Elmer; Software: Harmony 5.1). LAMP1-positive spots were identified and GCase-/hIgG-positive spots within LAMP1-positive spots were counted and normalised to total LAMP1-positive spots.

To assess potency of the GCase-BS molecules over time, cells were seeded at different starting densities (ranging from 4E4 cells/well for early time points to 1E4 cells/well for the last time point) into a 96-well plate and maintained at 37 °C, 5% CO2, 85% humidity for 16–18 h. Cells were then treated with a range of concentrations of the various GCase-BS molecules for 2 h. After incubation, cells were washed once with PBS and maintained in media for indicated times (0 h, 3 h, 6 h, 24 h, 48 h or 72 h). Subsequently, cells were washed once again with PBS and dry cell layer was frozen at −80 °C. Cells were then processed for GlcSph measurements as described in the main article.

### Enzymatic hydrolysis of GlcCer and GlcSph by imiglucerase and hGCase-BS

All reactions were incubated for 30 min at 37 °C in buffered solution (0.05 M citric acid, 0.05 M KH_2_PO_4_, 0.05 M K_2_HPO_4_, 0.11 M KCl, 0.01 M NaCl, 0.001 M MgCl_2_) at pH 6.0.

Liquid chromatography-mass spectrometry analysis of GlcSph and GlcCer was done as described in the main article. Data reflect qualitative measurements only. Reactant intensity was set to 100% for each reaction. Product intensity was normalised to overall highest intensity.

### Liquid chromatography-mass spectrometry analysis of GlcSph

Analytes and internal standards were purchased from Avanti Polar Lipids: D-glucosyl-β−1-1’-D-erythro-sphingosine (GlcSph (d18:1); #860535) and D-glucosyl-β−1-1’-D-erythro-sphingosine-d5 as internal standard 1 (GlcSph-d5 (d18:1); #860636); D-galactosyl-β−1-1’-D-erythro-sphingosine (GalSph (d18:1); #860537) and D-galactosyl-β−1-1’-D-erythro-sphingosine-d5 as internal standard 2 (GalSph-d5 (d18:1); #860637).

For chromatography HPLC grade solvents as well as Millipore water was used. Acetonitrile (LiChrosolv #1.00030) and methanol (LiChrosolv #1.06007) were obtained from Supelco (Merck), ammonium acetate for mass spectrometry was purchased by Sigma-Aldrich (#73594).

Stock solutions for analytes and internal standards were prepared at 1 mM in DMSO and kept at −20 °C. For further spiking solutions acetonitrile/water 9/1 (v/v) was used as solvent. Calibration solutions were prepared by serial dilution in acetonitrile/water 9/1 (v/v) containing 2% DMSO. The concentration range was from C1 = 10 µM to C9 = 0.0039 µM. Final calibration samples were made in a pooled tissue homogenate and prepared exactly the same way as tissue samples in order to avoid suppression effects deriving from the biological matrix.

Analysis was conducted on a LC-MS-MS system consisting of a Waters Xevo-TQ-S mass spectrometer connected to a complete Waters Acquity I-class UPLC system with a flow through needle sample manager using a mixture of acetonitrile/methanol/water 40/40/20 (v/v/v) as wash solvent. The auto-sampler temperature was set to 15 °C. The Xevo TQ-S instrument operated in positive ion electrospray mode with both quadrupoles tuned to unit mass resolution using nitrogen as nebulization- and desolvation gas. The nebulizer gas flow was set to 150 l/h and the desolvation gas flow to 800 l/h with a temperature of 500 °C. Argon was used as collision gas at a flow rate of 0.15 ml/min. Analytes and internal standards were detected by multiple reaction monitoring mode (MRM) following the transitions m/z 462.3 to 282.3 and m/z 467.3 > 287.3 at a cone voltage of 30 V and a collision energy of 18 V.

Samples were analysed on a BEH glycan amide column (100 × 2.1 mm, 1.7 µm particle size, purchased from Waters, Switzerland) with a flow rate of 0.25 ml/min and an oven temperature of 30 °C. Eluent A consisted of 100 mM ammonium acetate and for eluent B acetonitrile was used. Glycospecific separation was achieved by isocratic elution with 90% B followed by a washing step with 10% B and column reconditioning. The overall analysis time was 12 min.

Data acquisition and analysis was done using MassLynx V4.1 SCN950 (Copyright 2015 Waters Inc.).

#### Sample preparation for in vitro GlcSph measurements

Deep frozen cell pellets were obtained in a 96 well cell culture plate. 50 µl of distilled water was added to each well. The plate was sealed and shaken for 30 min at 25 °C until the cells had thawed. Then, 100 µl methanol containing 0.005 µM internal standards was added. After shaking 5 min, the supernatant was transferred to a new plate. Each well of the cell culture plate was washed with another 100 µl of methanol. The samples were evaporated to dryness and reconstituted in 100 µl acetonitrile/water 90/10 (v/v) containing 1% DMSO and analysed by LC-MS/MS. Lipids were quantified using the peak area ratio analyte/internal standard (=response).

#### Sample preparation for in vivo GlcSph measurements

Frozen tissues were weighed into 7 ml hard tissue homogenising vials prefilled with ceramic beads (Bertin Cat.No.03961-1-002.2 (CK28), supplied by LabForce AG, Switzerland or from Omni International, CatNo.19-628) and homogenised with distilled water giving a final concentration of 100 mg tissue/ml. Samples, QC’s and calibration samples were cleaned up by protein precipitation with methanol containing internal standards. After centrifugation the supernatants were evaporated to dryness, reconstituted in acetonitrile/water 90/10 (v/v) with 1% DMSO and analysed by LC-MS/MS. For calibration, a linear regression function with 1/y weighting and excluding zero was used. The calibration range was from x + 39 nM (C9) to x + 1 µM (C1), where x is the endogenous substrate level in the pooled tissue homogenate. Absolute concentration was calculated by dividing the peak area ratio analyte/internal standard by the slope of the calibration curve.

### Liquid chromatography-mass spectrometry analysis of GlcCer

Analytes and internal standards were purchased from Avanti Polar Lipids or Cayman Chemicals.

D-glucosyl-ß−1,1’-N-palmitoyl-D-erythro-sphingosine (GlcCer (d18:1/16:0); #860539), D-glucosyl-ß−1,1’-N-stearoyl-D-erythro-sphingosine (GlcCer (d18:1/18:0); #860547), D-glucosyl-ß−1,1’-N-nervonoyl-D-erythro-sphingosine (GlcCer (d18:1/24:1); #860549), D-galactosyl-ß−1,1’ N-palmitoyl-D-erythro-sphingosine (GalCer (d18:1/16:0); #560521), D-galactosyl-ß−1,1’-N-stearoyl-D-erythro-sphingosine (GalCer (d18:1/18:0); #860844), D-galactosyl-ß−1,1’ N-nervonoyl-D-erythro-sphingosine (GlcCer (d18:1/24:1); #860546) and D-glucosyl-ß−1,1’-N-stearoyl-D-erythro-sphingosine-d5 (GlcCer-d5 (d18:1/18:0 #860638)) as internal standard 1 were purchased from Avanti Lipids.

N-Docosanoyl-β-glucosylsphingosine (GlcCer (d18:1/22:0); #23210), N-Docosanoyl-β-galactosylsphingosine (GalCer (d18:1/22:0); #9003458) and N-Docosanoyl-β-glucosylsphingosine-d4 (GlcCer-d4 (d18:1/22:0); #9003462) as internal standard 2 were purchased from Cayman Chemicals.

For chromatography HPLC grade solvents as well as Millipore water was used. Acetonitrile (LiChrosolv #1.00030) and methanol (LiChrosolv #1.06007) were obtained from Supelco, ammonium formate for mass spectrometry was purchased by Sigma-Aldrich (#70221) and formic acid HiPerSolv CHROMANORM® for LC-MS was obtained from VWR chemicals (#84865.180).

Stock solutions for analytes and internal standards were prepared with a concentration of 1 mM in DMSO and kept at −20 °C. For further spiking solutions acetonitrile/water 9/1 (v/v) was used as solvent.

Analysis was conducted on a LC-MS-MS system consisting of a Waters Xevo-TQ-S mass spectrometer connected to a complete Waters Acquity I-class UPLC system with a flow through needle sample manager using a mixture of acetonitrile/methanol/water 40/40/20 (v/v/v) as wash solvent. The auto-sampler temperature was set to 15 °C. The Xevo TQ-S instrument operated in positive ion electrospray mode with both quadrupoles tuned to unit mass resolution using nitrogen as nebulization- and desolvation gas. The nebulizer gas flow was set to 150 l/h and the desolvation gas flow to 800 l/h with a temperature of 500 °C. Argon was used as collision gas at a flow rate of 0.15 ml/min. Analytes and internal standards were detected by multiple reaction monitoring mode (MRM) following the transitions m/z 700.6 > 264.3 (HexCer 16:0), 728.6 > 264.3 (HexCer 18:0), 784.6 > 264.3 (HexCer 22:0), 810.7 > 264.3 (HexCer 24:1), 733.6 > 269.3 (GlcCer 18:0-d5) and 788.6 > 264.3 (GlcCer 22:0-d4) using a cone voltage of 30 V and a collision energy of 30 or 40 V.

Samples were analysed on an Atlantis Hilic Silica column (150 × 2.1 mm, 3 µm particle size, purchased from Waters Switzerland) with a flow rate of 0.5 ml/min and an oven temperature of 40 °C. Eluent A consisted of an aqueous solution of 5 mM ammonium formate and 0.05% formic acid and for eluent B a mixture of acetonitrile/methanol /water 95/2.5/2.5 (v/v/v) with 5 mM ammonium formate and 0.05% formic acid was used. Glycospecific separation was achieved by isocratic elution with 100% B followed by a washing step with 50% A and column reconditioning. The overall analysis time was 7 min.

#### Sample preparation for in vitro GlcCer measurements

Deep frozen cell pellets were obtained in a 96 well cell culture plate. 50 µl of distilled water was added to each well. The plate was sealed and shaken for 30 min at 25 °C until the cells have been thawed. Then 100 µl methanol containing 0.01 µM internal standard was added. After shaking another 5 min. the supernatant was transferred to a new plate. Each well of the cell culture plate was washed with another 100 µl of methanol. The samples were evaporated to dryness and reconstituted in 100 µl acetonitrile containing 1% DMSO and analysed by LC-MS/MS. Lipids were quantified using the peak area ratio analyte/internal standard (=response).

### Assessment of pharmacokinetics and -dynamics of GCase-BS in vivo

C57BL/6 male mice were dosed i.v. with 2.5 mg/kg of mGCase-mBS and blood or tissue samples were taken at defined time points after dosing (blood: 5 min, 15 min, 1 h, 4 h, 7 h and 24 h; brain: 24 h, 48 h and 72 h). Blood was collected in EDTA-K2 tubes and centrifuged at 1000 x g for 10 min at 4 °C to obtain plasma. Brain samples were snap frozen upon preparation and mechanically homogenised in 500 μL of tissue extraction buffer containing protease inhibitors using the MagNA Lyser Homogenisator.

To determine plasma or brain tissue IgG concentrations, samples were analysed with a generic ECLIA method specific for the human Ig/Fab CH1/kappa domain using the cobas e411 instrument under non-GLP conditions. In brief, samples, primary detection antibody (mAb anti-hFab(kappa)), secondary detection antibody (mAb anti-hFab(CH1)) and SA-beads were added stepwise to a detection vessel and incubated for 9 min in each step. Finally, the SA-beads-bound complex was detected by a measuring cell, which numbers the counts of SA-beads in repeat. The counts were proportional to the analyte concentration in the test sample.

To determine the activity of mGCase-mBS in plasma samples, a capture assay was performed using a mAb anti-hFab(kappa) as capture antibody. After subsequent washes, GCase enzymatic activity was determined using Amplex™ Red Glucose/Glucose Oxidase Assay Kit (Thermo, #A22189). A defined standard curve of active mGCase-mBS in the same matrix was used to determine amounts of active compound in plasma over time.

### NFL analysis in mouse plasma

NFL detection in mouse plasma using Quanterix’ digital biomarker detection technology, Simoa®:

38 µl mouse plasma was mixed with 152 µl sample diluent from NF-light Kit (Quanterix #103186) and processed according to the manufacturers’ instructions.

### Statistical analysis

Statistical comparison of data was done using GraphPad Prism 8.4.2. Parametric tests (Student’s two-tailed *t*-test for pairwise comparisons or ANOVA for multiple comparisons) were used and are indicated for each experiment in the respective figure legend. The *p* values and n values for all comparisons are indicated in each figure legend. For in vitro studies, if not stated otherwise, *n* = number of independent measurements. For in vivo studies, *n* = number of animals per group.

### Immunoprecipitation of lysosomes (Lyso-IP method) for proteomic and lipidomic analysis

H4 cells (*GB*+/+ and *GBA−/−*) stably expressing TMEM192-3XHA were seeded in 10/15 cm cell culture dish such that sufficient cells (≅ 2E7 cells for proteomics and ≅ 5E7 cells for lipidomics/replicate) are available on the day of lysosome isolation. *GBA−/−* cells were treated with 1 nM hGCase-hBS for 24 h. On the day of lysosome isolation, cells were washed with ice-cold PBS, gently scraped, and centrifuged at 1000 x g for 2 min. Cell pellets were resuspended in 1000 µl of ice-cold PBS and gently lysed using a rotary dounce homogeniser at medium speed. Homogenate was centrifuged at 1000 x g for 2 min to remove cell debris. Part of the supernatant was preserved for quality control analysis. The remaining supernatant (≅ 900 µl) was incubated with 500 ul of anti-HA magnetic beads (Pierce/Thermo: 88836/88837) for 20 min at room temperature in a rotator shaker. Magnetic beads were separated using a magnetic rack and the flow-through was collected for quality control analysis. The magnetic beads carrying lysosomes were washed with ice-cold PBS. For the proteomic samples, 200 µl of 1X RIPA buffer was added to magnetic beads carrying lysosomes and heated for 5 min at 95 °C. The resulting protein samples from lysosomes were acetone precipitated and used for further analysis. For the lipidomic samples, lysosomes were separated from magnetic beads using competitive elution due to the presence of high concentration of HA peptide. Magnetic beads carrying lysosomes were incubated with 500 ul of 1 mg/ml HA peptide (in PBS) and incubated for 15 min at 37 °C. Magnetic beads were removed using a magnetic rack and the remaining lysosome containing samples were immediately frozen at −80 °C for further analysis. For corresponding whole-cell lysate samples, cells were seeded in 10 cm culture dish (≅ 8E6 cells per replicate) and treated with hGCase-hBS wherever applicable. Cells were gently scraped and cell pellets were collected by centrifugation at 1000 x g for 2 min.

### Proteomics sample preparation

Samples were denatured using Biognosys’ Denature Buffer, reduced using Biognosys’ Reduction Solution for 60 min at 37 °C and alkylated using Biognosys’ Alkylation Solution for 30 min at room temperature in the dark. Subsequently, digestion to peptides was carried out using 0.5 µg of trypsin (Promega) per sample overnight at 37 °C. Peptides were desalted using a C18 MicroSpin plate (The Nest Group) according to the manufacturer’s instructions and dried down using a SpeedVac system Peptides were resuspended in 20 µl LC solvent A (1% acetonitrile, 0.1% formic acid (FA)) and spiked with Biognosys’ iRT kit calibration peptides. Peptide concentrations were determined using a UV/VIS Spectrometer (SPECTROstar Nano, BMG Labtech).

### HRM mass spectrometry acquisition for proteomics

For DIA LC-MS/MS measurements, 1 µg of peptides per sample were injected to an in house packed reversed phase column (PicoFrit emitter with 75 µm inner diameter, 60 cm length and 10 µm tip from New Objective, packed with 1.7 µm Charged Surface Hybrid C18 particles from Waters) on a Thermo Scientific™ EASY-nLC ™ 1200 nano liquid chromatography system connected to a Thermo Scientific™ Q Exactive™ HF mass spectrometer equipped with a Nanospray Flex™ Ion Source. LC solvents were A: 1% acetonitrile in water with 0.1% FA; B: 20% water in acetonitrile with 0.1 % FA. The nonlinear LC gradient was 1−59% solvent B in 55 min followed by 59–90% B in 10 s, 90% B for 8 min, 90% − 1% B in 10 s and 1% B for 5 min at 60 °C and a flow rate of 250 nl/min. The DIA method consisted of one full range MS1 scan and 21 DIA segments adopted from Bruderer et al. ^73^.

### Proteomics data analysis

Proteins with low intensities and NA values were filtered, and subsequently analysed using the edgeR Bioconductor package. Protein name to HUGO gene symbol mapping was performed using Bioconductors org.Hs.eg.db package. Libraries were normalised using TMM to remove composition bias, and we fitted a negative binomial generalised log-linear model to the log2 intensities of each protein, taking into account genewise, trended and common dispersion estimates. Testing for differential expression of proteins between comparison groups was tested with a log likelihood ratio test. For comparison and ranking of interesting hits resulting from the contrasts of interest, we introduced a comparison metric which combines significance and change in protein abundance: metric = −Log10(*p*.value) * Log2(FC). A metric threshold of 6 was used for filtering the top differentially expressed proteins.

### Lipid extraction for mass spectrometry lipidomics

Mass spectrometry-based lipid analysis was performed by Lipotype GmbH (Dresden, Germany) as previously described^74–77^ Lipids were extracted using a two-step chloroform/methanol procedure^74^. Samples were spiked with internal lipid standard mixture containing: cardiolipin 14:0/14:0/14:0/14:0 (CL), ceramide 18:1;2/17:0 (Cer), diacylglycerol 17:0/17:0 (DAG), hexosylceramide 18:1;2/12:0 (HexCer), lyso-phosphatidate 17:0 (LPA), lyso-phosphatidylcholine 12:0 (LPC), lyso-phosphatidylethanolamine 17:1 (LPE), lyso-phosphatidylglycerol 17:1 (LPG), lyso-phosphatidylinositol 17:1 (LPI), lyso-phosphatidylserine 17:1 (LPS), phosphatidate 17:0/17:0 (PA), phosphatidylcholine 17:0/17:0 (PC), phosphatidylethanolamine 17:0/17:0 (PE), phosphatidylglycerol 17:0/17:0 (PG), phosphatidylinositol 16:0/16:0 (PI), phosphatidylserine 17:0/17:0 (PS), cholesterol ester 20:0 (CE), sphingomyelin 18:1;2/12:0;0 (SM), triacylglycerol 17:0/17:0/17:0 (TAG) and cholesterol D6 (Chol). After extraction, the organic phase was transferred to an infusion plate and dried in a speed vacuum concentrator. 1st step dry extract was re-suspended in 7.5 mM ammonium acetate in chloroform/methanol/propanol (1:2:4, V:V:V) and 2nd step dry extract in 33% ethanol solution of methylamine in chloroform/methanol (0.003:5:1; V:V:V). All liquid handling steps were performed using Hamilton Robotics STARlet robotic platform with the Anti Droplet Control feature for organic solvents pipetting.

### MS data acquisition for lipidomics

Samples were analysed by direct infusion on a QExactive mass spectrometer (Thermo Scientific) equipped with a TriVersa NanoMate ion source (Advion Biosciences). Samples were analysed in both positive and negative ion modes with a resolution of Rm/z = 200 = 280000 for MS and Rm/z = 200 = 17500 for MSMS experiments, in a single acquisition. MSMS was triggered by an inclusion list encompassing corresponding MS mass ranges scanned in 1 Da increments^76^. Both MS and MSMS data were combined to monitor CE, DAG and TAG ions as ammonium adducts; PC, PC O-, as acetate adducts; and CL, PA, PE, PE O-, PG, PI and PS as deprotonated anions. MS only was used to monitor LPA, LPE, LPE O-, LPI and LPS as deprotonated anions; Cer, HexCer, SM, LPC and LPC O- as acetate adducts and cholesterol as ammonium adduct of an acetylated derivative^77^.

### Lipidomics data analysis

Data were analysed with in-house developed lipid identification software based on LipidXplorer^78,79^. Data post-processing and normalisation were performed using an in-house developed data management system. Only lipid identifications with a signal-to-noise ratio > 5, and a signal intensity 5-fold higher than in corresponding blank samples were considered for further data analysis. Data were analysed with R version 4.0.3 (2020-10-10) (RCore Team 2017) using tidyverse packages (version 1.3.0)^80^ and bioconductor pcaMethods^81^. Lipids were quantified in molar fractions (molp) and standardised to the total lipid amount per sample due to large differences in the total lipid amounts across samples. A 70% occupational threshold was applied, yielding 1196 lipids to be compared. Differential lipidomics analysis was performed using an unpaired t-test between the test groups (GBA-KO vs. WT and KOE vs. KO in both lysosomal and whole cell lysates). Fold changes between comparison groups are defined as the Log2 fold change of the means. We used the same combined p-value/logFC metric as for the lipid contrasts to compare the top hits. For displaying the top hits between GBA-KO vs. WT and KOE vs. KO comparison (Fig. 5d, Supplementary Fig. 7), we applied a metric threshold of 1 for both directions.

### iPSC-derived M0 macrophages

hiPSC-derived M0 macrophages differentiation was performed as previously described^82^. Briefly, hiPSCs were maintained in mTeSR Plus (Cat# 100-0276, STEMCELL Technologies Inc.) media at 37 °C and 5% CO_2_. Culture vessels were coated with 12.5 µg/mL of rhLaminin-521 (Biolaminin LN, Cat# LN521, BioLamina AB) in PBS with Calcium and Magnesium (PBS+/+, Cat# 14040141, Thermo Fisher Scientific) for 2 h at RT prior seeding of the cells. To differentiate M0 macrophages, embryoid bodies (EBs) were formed by detaching hiPSCs from the plates using Accutase (Cat# AT104, Innovative Cell Technologies, Inc.) to obtain a single-cell suspension. 4E6 cells were plated per well in a 24-well micro-well plate (AggreWell 800, Cat# 34811, STEMCELL Technologies Inc.) in 2 mL of mTeSR Plus supplemented with 10 µM ROCK inhibitor (Y-27632, Cat# SCM075, Merck KGaA) and the plates were centrifuged at 100 x g for 3 min. In the following 3 days, 75% of the media was replaced in each well with mTeSR Plus containing 50 ng/mL human Bone morphogenetic protein 4 (hBMP4) (Cat# 314-BP, R&D Systems Inc.), 50 ng/mL human vascular endothelial growth factor (hVEGF) (Cat# 293-VE, R&D Systems Inc.), and 20 ng/mL human stem cell factor (hSCF) (Cat# 255-SC, R&D Systems Inc.). At day 4, the EBs were collected and plated in flasks pre-coated with growth factor reduced Matrigel (Cat# 354230, Corning Inc.) at 300 µg/mL in cold DMEM/F12 1:1 (Cat# 12634010, Thermo Fisher Scientific) for 1 h at RT at a density of 1.5 EB/cm2 in X-VIVO 15 (Cat# BE02-053Q, Lonza Bioscience) media containing 2 mM Glutamax (Cat# 35050061, Thermo Fisher Scientific), 100 U/mL penicillin/streptomycin, 50 µM 2-mercaptoethanol, 100 ng/mL M-CSF (Cat# 130-096-492, Miltenyi Biotec), and 25 ng/mL IL-3 (Cat# 130-095-07, Miltenyi Biotec) at 37 °C and 5% CO_2_. 50% media change was performed once a week for 2–3 weeks until the production and release of CD14 + macrophage progenitors in the media was achieved. During the following 6–8 weeks, macrophage precursor cells could be harvested from the media during the two weekly 100% media changes. For the differentiation to M0 macrophages, precursor macrophages were plated at a density of 1.2E5 cells/cm² in X-VIVO 15 (supplemented with 2 mM Glutamax, 1% pen/strep, and 100 ng/mL M-CSF) in 96-well plates pre-coated with 10 µg/mL of Fibronectin (Cat# 356008, Corning Inc.) in PBS+/+ for 2 h at RT. To obtain M0 macrophages, the cells were differentiated for 9 days and 50% media-change performed at day 3, 5 and 7. Cells were treated with the indicated concentrations of imiglucerase or hGCase-hBS for 9 days prior the assay. For GlcSph analysis, cells were washed once in cold PBS and dry cell layer was frozen at −80 °C. For the assessment of GCase enzymatic activity, cells were washed once in cold PBS and lysed by pipetting up and down for 30 times with 40 µl/well of lysis buffer (50 mM citric acid, 50 mM KPi, 110 mM KCl, 10 mM NaCl, 1 mM MgCl_2_, 0.25% v/v Triton X-100 at pH 6.0) supplemented with protease inhibitor cocktail (cOmplete, Roche) as described by the manufacturer before storing the plates at −80 °C.

### Human mannose receptor detection in human neurons, H4 or macrophages

To determine human mannose receptor (MR) protein levels in cells, an ELISA kit (Abcam, #ab277420) was used according to the manufacturer’s instructions. In brief, 1E6 cells of differentiated human neurons, H4 cells, or macrophages were collected and lysed in 1X RIPA buffer. Subsequently, samples were subjected to serial dilution (F = 3) and incubated with capture antibody for 2.5 h at room temperature. After washing, biotinylated detection antibody was incubated for 1 h at room temperature. Finally, signal was measured upon chromogenic detection by HRP-TMB reaction. Human MR protein levels in samples were extrapolated from a standard curve.

### Reporting summary

Further information on research design is available in the Nature Portfolio Reporting Summary linked to this article.

## Supplementary information

Supplementary Information

Description of Additional Supplementary Files

Supplementary Data 1

Supplementary Data 2

Supplementary Data 3

Reporting Summary

## Data Availability

The mass spectrometry proteomics data have been deposited to the ProteomeXchange Consortium via the PRIDE partner repository with the dataset identifier PXD036361. All other source data are provided with this paper in the Source Data file. Source data are provided with this paper.

## Source data

Source Data
