# Supplementary material for: Targeting neuronal lysosomal dysfunction caused by β-glucocerebrosidase deficiency with an enzyme-based brain shuttle construct
Source: Nat Commun. 2023 Apr 12;14:2057. doi: 10.1038/s41467-023-37632-4 (PMC10097658; doi:10.1038/s41467-023-37632-4)
Supplement: Supplementary file 1 — Supplementary Information [file 41467_2023_37632_MOESM1_ESM.pdf]

Targeting neuronal lysosomal dysfunction caused by β-glucocerebrosidase deficiency with an enzyme-based Brain Shuttle construct

Alexandra Gehrlein[1*], Vinod Udayar[1], Nadia Anastasi[1], Martino L. Morella[1,2], Iris Ruf[3], Doris Brugger[3], Sophia von der Mark[3], Ralf Thoma[3], Arne Rufer[3], Dominik Heer[3], Nina Pfahler[3,4], Anton Jochner[5], Jens Niewoehner[5], Luise Wolf[6], Matthias Fueth[7], Martin Ebeling[7], Roberto Villaseñor[1], Yanping Zhu[8], Matthew C. Deen[8], Xiaoyang Shan[8], Zahra Ehsaei[9], Verdon Taylor[9], Ellen Sidransky[10], David J. Vocadlo[8,11], Per-Ola Freskgård[1,12], Ravi Jagasia[1*]

[1]Roche Pharma Research and Early Development, Neuroscience and Rare Diseases Discovery and Translational Area, Roche Innovation Center Basel, F. Hoffmann-La Roche Ltd, Basel, Switzerland
[2]Department of Anatomy and Neurosciences, Amsterdam University Medical Center | VUmc, Amsterdam, Netherlands
[3]Roche Pharma Research and Early Development, Therapeutic Modalities, Lead Discovery, Roche Innovation Center Basel, F. Hoffmann-La Roche Ltd, Basel, Switzerland
[4]Interfaculty Institute of Biochemistry & Structural Biology Biochemistry (IFIB), Eberhard Karls University of Tübingen, Germany
[5]Roche Pharma Research and Early Development, Therapeutic Modalities Large Molecule Research, Roche Innovation Center Munich, Roche Diagnostics GmbH, Penzberg, Germany
[6]Roche Pharma Research and Early Development, Data & Analytics, Roche Innovation Center Basel, F. Hoffmann-La Roche Ltd, Basel, Switzerland
[7]Roche Pharma Research and Early Development, Pharmaceutical Science, Roche Innovation Center Basel, F. Hoffmann-La Roche Ltd, Basel, Switzerland
[8]Department of Chemistry, Simon Fraser University, Burnaby, British Columbia V5A 1S6, Canada
[9]Department of Biomedicine, University of Basel, Basel, Switzerland
[10]Molecular Neurogenetics Section, National Human Genome Research Institute, Bethesda, US
[11]Department of Molecular Biology and Biochemistry, Simon Fraser University, Burnaby, British Columbia V5A 1S6, Canada
[12]BioArctic AB, Stockholm, Sweden

*Corresponding authors:
ravi.jagasia@roche.com
alexandra.gehrlein@roche.com

SUPPLEMENTARY INFORMATION

Supplementary Table 1. Enzymatic properties of relevant GCase(-BS) molecules. Values in parentheses are 95% confidence intervals of the fitted parameters. Source data are provided as a Source Data file.

**Construct**	**k_{cat} [s^{-1}]**	**K_M [µM]**	**k_{cat}/K_M [M^{-1} * s^{-1}]**
Imiglucerase (CHO)	0.13<br>(0.12 to 0.14)	138<br>(112 to 171)	928
hGCase-NB	0.13<br>(0.11 to 0.16)	138<br>(102 to 192)	938
hGCase-hBS	0.32<br>(0.26 to 0.42)	66<br>(37 to 120)	4853
mGCase	0.14<br>(0.13 to 0.16)	86<br>(70 to 105)	1663
mGCase-mBS	0.25<br>(0.23 to 0.27)	114<br>(95 to138)	2151

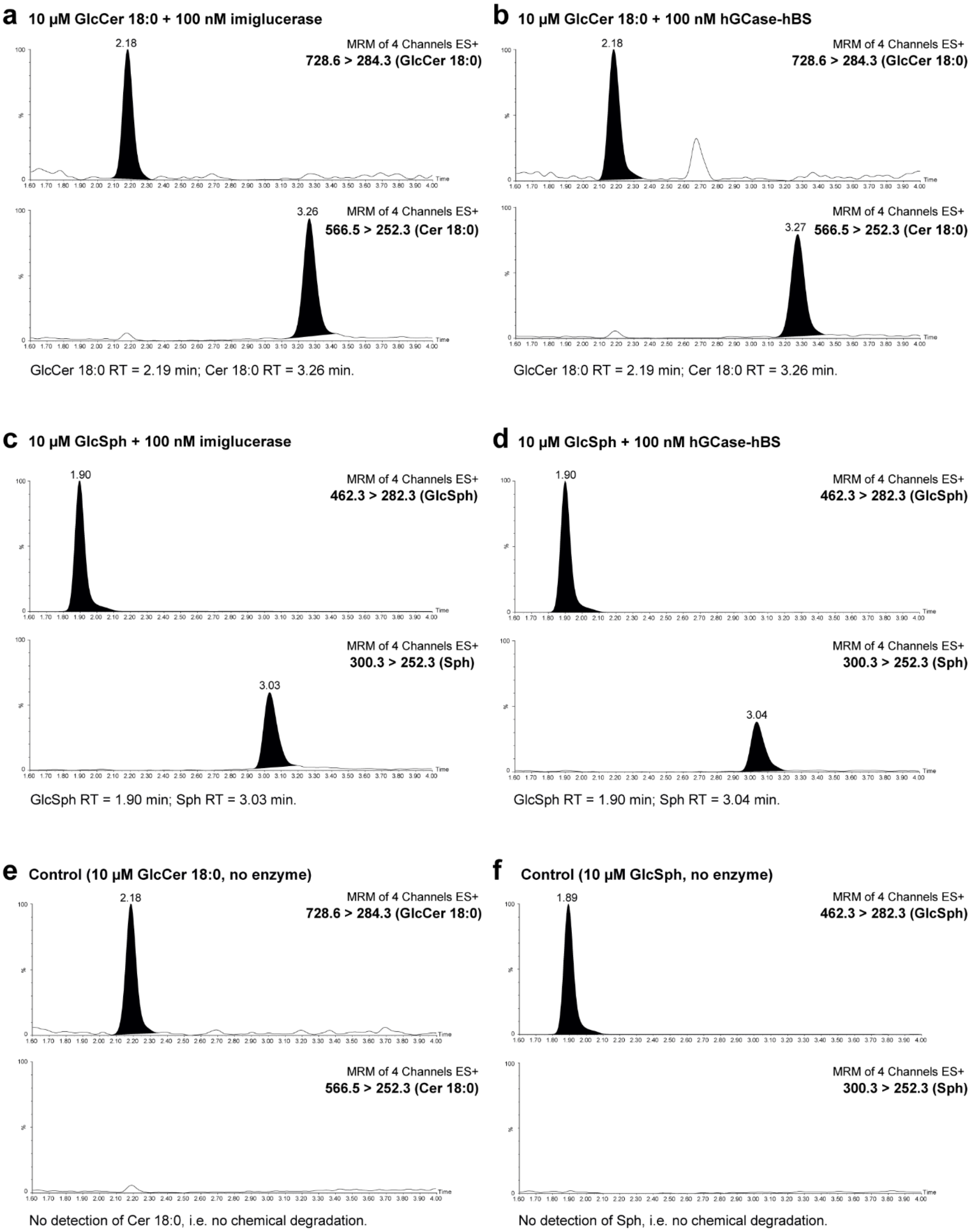

Supplementary Fig. 1. Enzymatic hydrolysis of GlcCer and GlcSph by imiglucerase and hGCase-BS.

a Chromatogram of reaction of 10 µM of GlcCer 18:0 with 100 nM of imiglucerase. Endpoint detection of reactant (m/z 728.6 > 284.3 (GlcCer 18:0)) and product (m/z 566.5 > 252.3 (Cer 18:0)) at t = 30 min. **b** Chromatogram of reaction of 10 µM of GlcCer 18:0 with 100 nM of hGCase-hBS. Endpoint detection of reactant (m/z 728.6 > 284.3 (GlcCer 18:0)) and product (m/z 566.5 > 252.3 (Cer 18:0)) at t = 30 min. **c** Chromatogram of reaction of 10 µM of GlcSph with 100 nM of imiglucerase. Endpoint detection of reactant (m/z 462.3 > 282.3 (GlcSph) and

product (m/z 300.3 > 252.3 (Sph)) at t = 30 min. **d** Chromatogram of reaction of 10 µM of GlcSph with 100 nM of hGCase-hBS. Endpoint detection of reactant (m/z 462.3 > 282.3 (GlcSph) and product (m/z 300.3 > 252.3 (Sph)) at t = 30 min. **e** Chromatogram of control setup with 10 µM of GlcCer 18:0 without enzyme. No product was detected at m/z 566.5 > 252.3 (Cer 18:0) at t = 30 min. **f** Chromatogram of control setup with 10 µM of GlcSph without enzyme. No product was detected at m/z 300.3 > 252.3 (Sph) at t = 30 min.

All reactions were incubated for 30 min. at 37 °C in buffered solution at pH 6.0. Data reflect qualitative measurements only. Reactant intensity was set to 100% for each reaction. Product intensity was normalised to overall highest intensity.

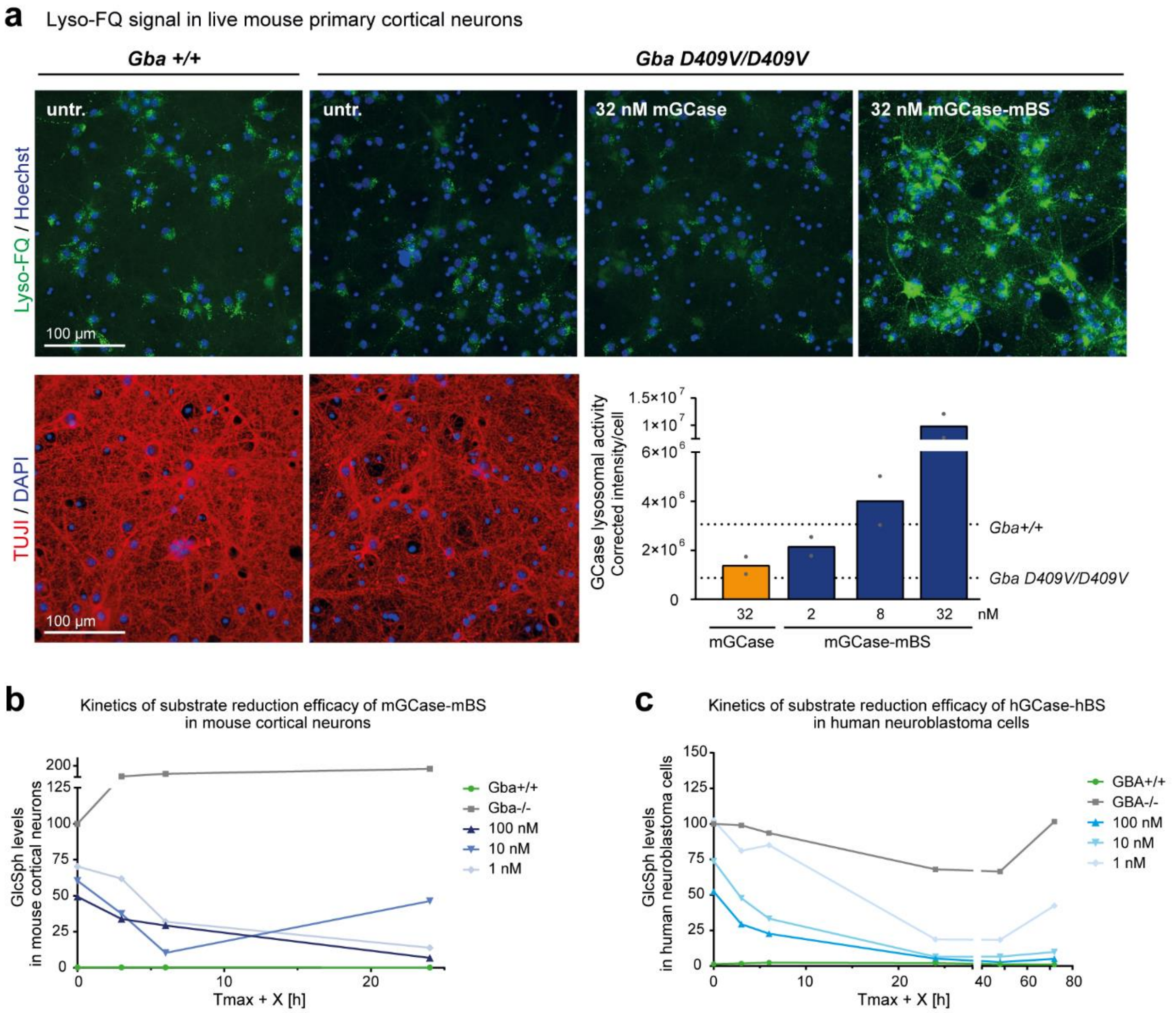

Supplementary Fig. 2. Additional *in vitro* experiments showing lysosomal activity and sustained efficiency of GCase-BS molecules.

a Lysosomal activity of mGCase vs. mGCase-mBS in primary cortical neurons from *Gba1 D409V/D409V* mice. n = 2. **b** Kinetics of GlcSph reduction upon 2 h treatment with mGCase-mBS in *Gba-/-* mouse neurons. n = 1. **c** Kinetics of GlcSph reduction upon 2 h treatment with hGCase-hBS in *GBA-/-* H4 cells. n = 1.

Data are represented as mean of technical replicates. n = number of independent measurements. Source data are provided as a Source Data file.

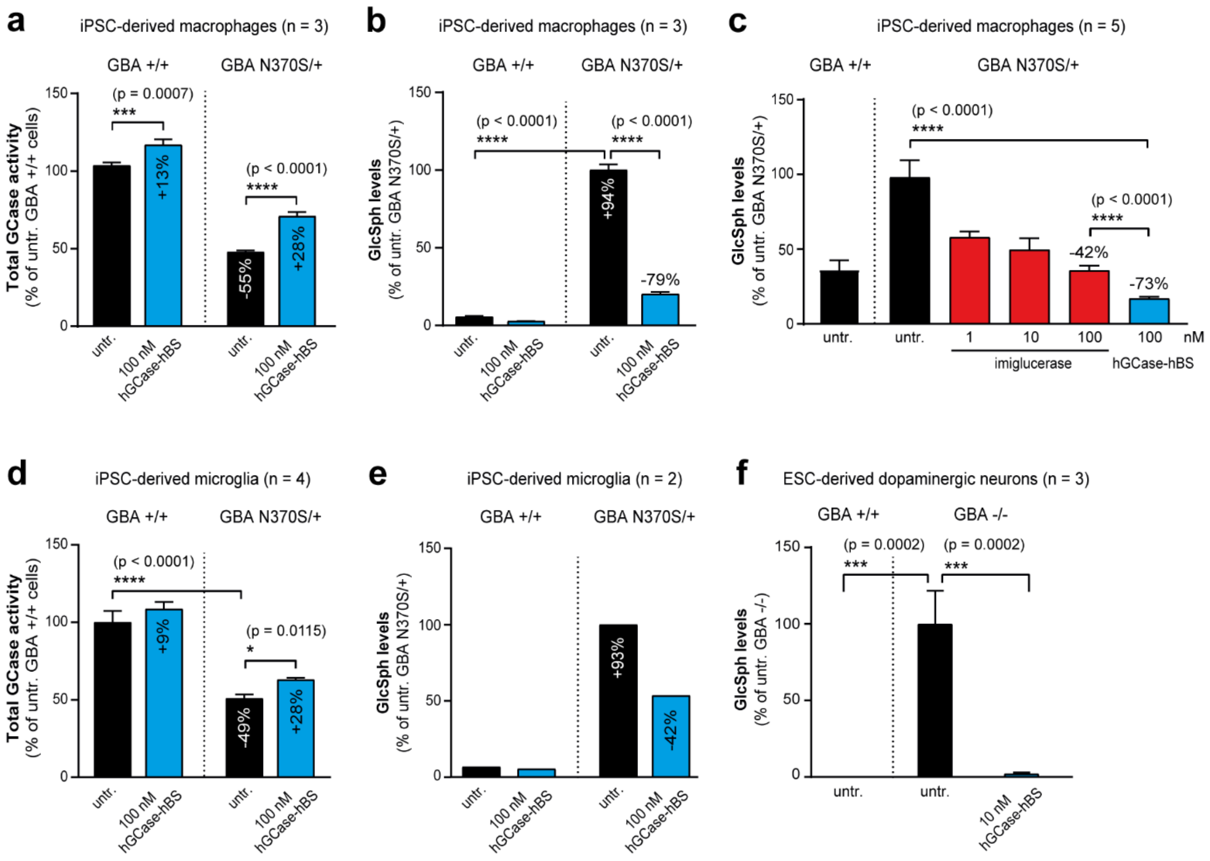

Supplementary Fig. 3. Uptake and lysosomal efficacy of hGCase-hBS in iPSC-derived macrophages and microglia or ESC-derived dopaminergic neurons.

a Total GCase activity upon 9 days of treatment with hGCase-hBS in iPSC-derived *GBA+/+* and *GBA N370S/+* macrophages. n = 3. **b** GlcSph levels upon 9 days treatment with hGCase-hBS in iPSC-derived *GBA+/+* and *GBA N370S/+* macrophages. n = 3. **c** Comparison of GlcSph reduction efficacy of imiglucerase and hGCase-hBS levels upon 9 days treatment with hGCase-hBS in iPSC-derived *GBA+/+* and *GBA N370S/+* macrophages. n = 5. **d** Total GCase activity upon 9 days of treatment with hGCase-hBS in iPSC-derived *GBA+/+* and *GBA N370S/+* microglia. n = 4. **e** GlcSph levels upon 9 days treatment with hGCase-hBS in iPSC-derived *GBA+/+* and *GBA N370S/+* microglia. n = 2. **f** GlcSph measurement in ESC-derived dopaminergic neurons as a measure of efficacy after treatment with 10 nM of hGCase-hBS. Data was normalised to *GBA-/-* cells. n = 3.

Bar graphs represent group means + SD. n = number of independent measurements. Data obtained from macrophage and microglia experiments were analysed by two-way ANOVA (Tukey's multiple comparisons test). Data obtained from experiments using dopaminergic neurons were analysed by one-way ANOVA (Dunnett's multiple comparisons test). * p < 0.05; ** p < 0.01; *** p < 0.001; **** p < 0.0001. Source data are provided as a Source Data file.

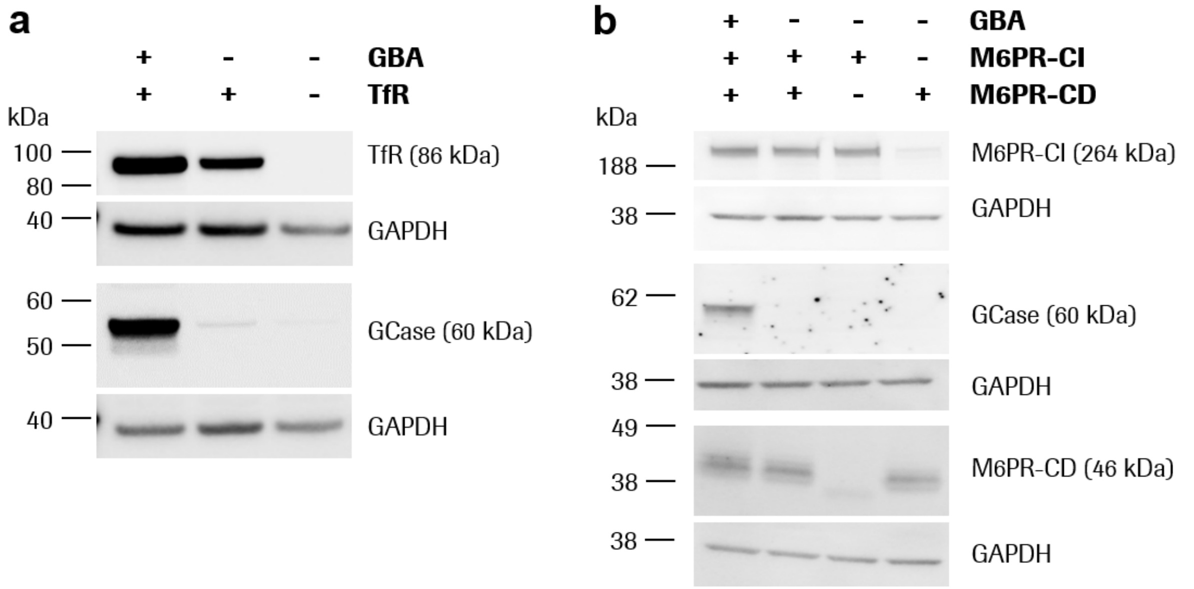

Supplementary Fig. 4. Confirmation of KO H4 cell lines by Western blot.

a Western Blot analysis to confirm the absence of GCase and TfR protein in double KO H4 line. **b** Western Blot analysis to confirm the absence of GCase and M6PR-CI or GCase and M6PR-CD protein in respective H4 KO lines.

10 µg of total protein lysate were loaded per lane. Representative blots of 3 replicates. Molecular weight is indicated in kilodalton (kDa).

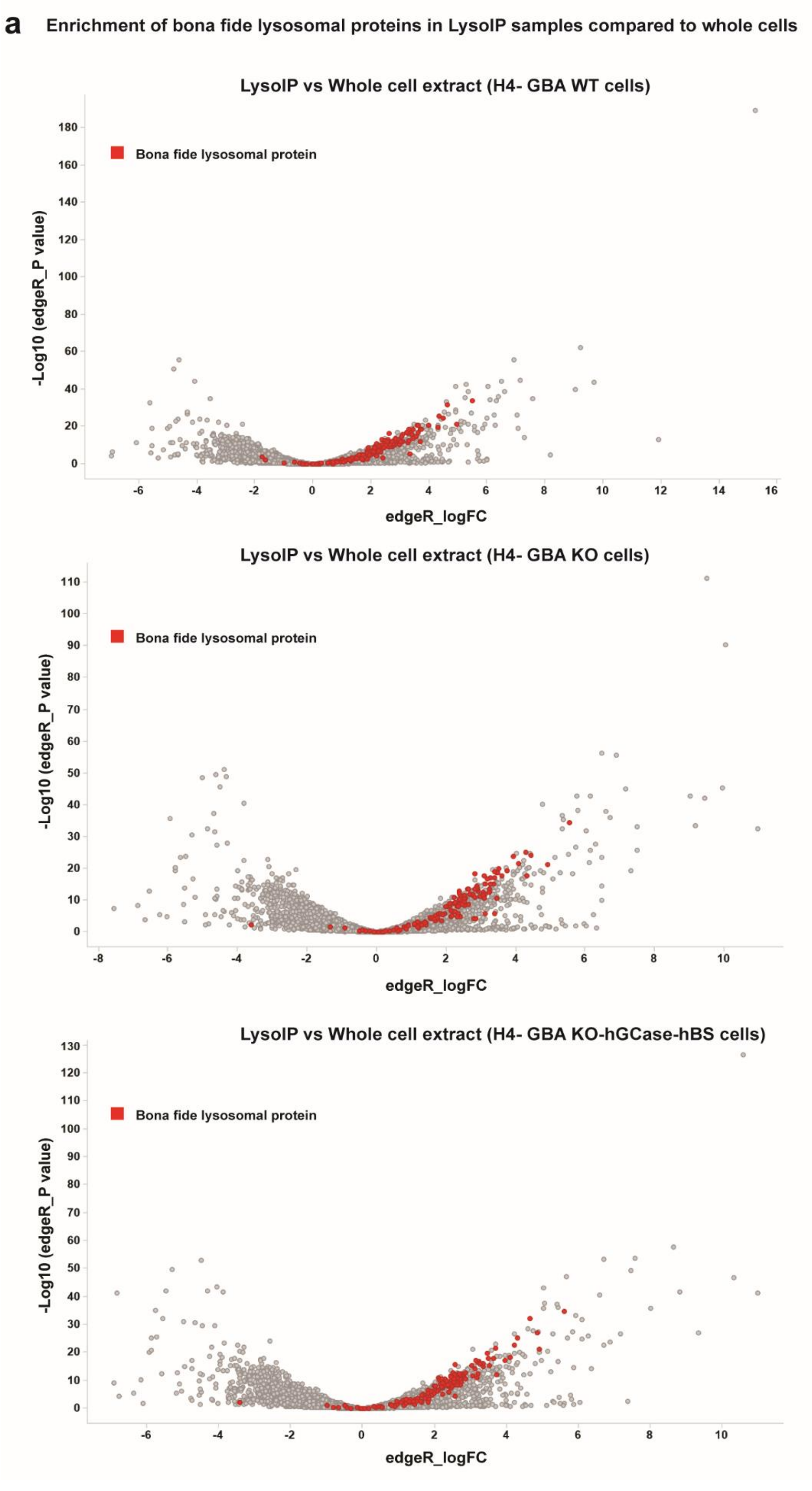

Supplementary Fig. 5. Enrichment of bona fide lysosomal proteins in isolated lysosomes.

a Volcano plot showing enrichment of bona fide lysosomal proteins (red dot) in Lyso-IP sample (lysosomes) compared to whole-cell extract. n = 3 biological replicates. Source data are provided as a Source Data file.

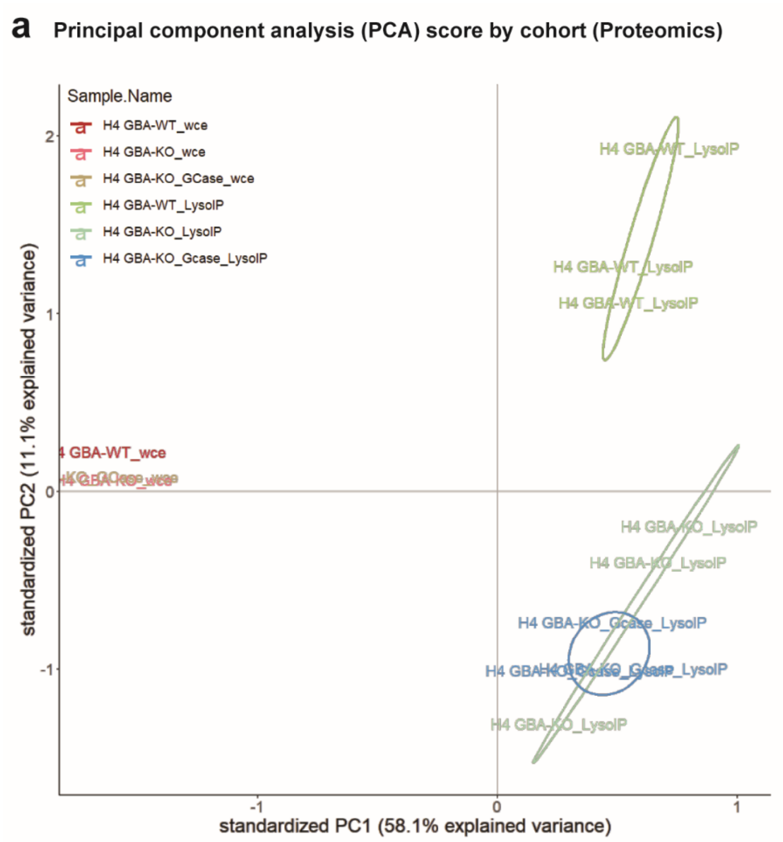

Supplementary Fig. 6. Principal component analysis (PCA) shows clear separation between lysosomal and whole-cell proteome.

a Principal component analysis was performed to visualise data set variance in dependence on sample groups. Main variance (PC 1) in this data set is explained by the difference between whole cell extracts (WCE) and lysosomes (LysoIP). Secondary variance (PC 2) in this data set is explained by the difference between wildtype lysosomes (WT_LysoIP) and GBA KO lysosomes (GBA KO_LysoIP). n = 3 biological replicates.

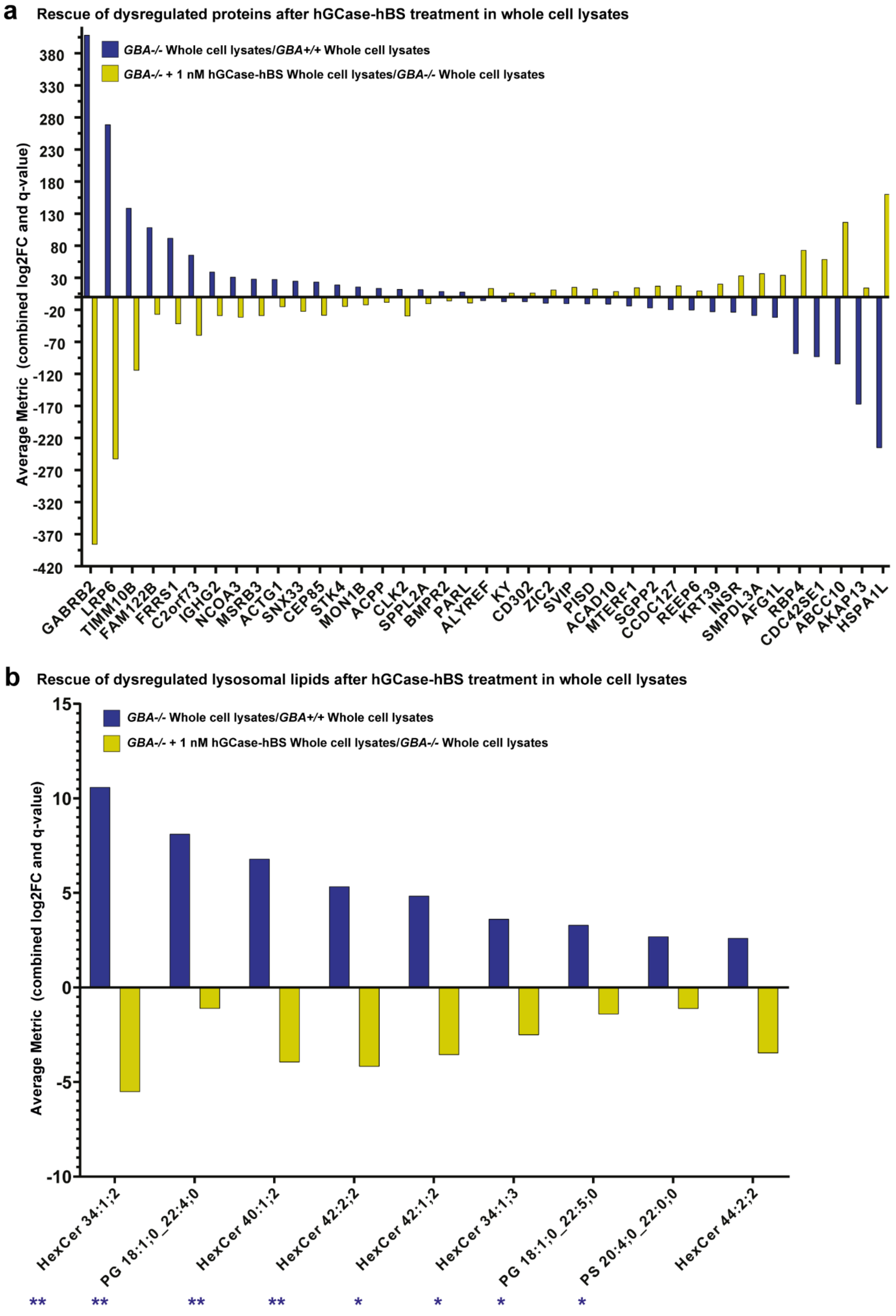

Supplementary Fig. 7. hGCase-hBS reverts protein and lipid dysregulation in whole-cell extract from *GBA1-/-* H4 cells.

a Rescue of dysregulated proteins upon hGCase-hBS treatment in whole-cell extract. Graph showing increased or decreased levels of proteins in *GBA1-/-* whole-cell extract (blue bar) and its rescue upon hGCase-hBS treatment (yellow bar). The majority of proteins displayed has a significant fold change (q-value < 0.05) in both contrasts (exceptions are MON1B/PARL/SGPP). **b** Rescue of dysregulated lipids upon hGCase-hBS treatment in whole-cell extract. Graph showing increased or decreased levels of lipid species in *GBA1-/-* whole-cell extract (blue bar) and its rescue upon hGCase-hBS treatment (yellow bar). Note: The lipid analysis performed does not report the number of carbons in the sphingoid base and the acyl chain (fatty acid chain) separately. Hence, the first number in the nomenclature used refers to the total number of carbons (sphingoid base + acyl chain). An Excel sheet consisting of each of the detected lipid species and its corresponding Swisslipids ID is provided in Supplementary Data File 3.

FDR-corrected p-values are shown (** q-value < 0.01; * q-value < 0.05; n = 3 biological replicates), calculated for each contrast separately. We are also displaying trends (e.g. significance found in one contrast only), to highlight potential lipid/protein entities with opposite fold change in *GBA-/-* and rescue upon hGCase-hBS treatment. Source data are provided as a Source Data file.

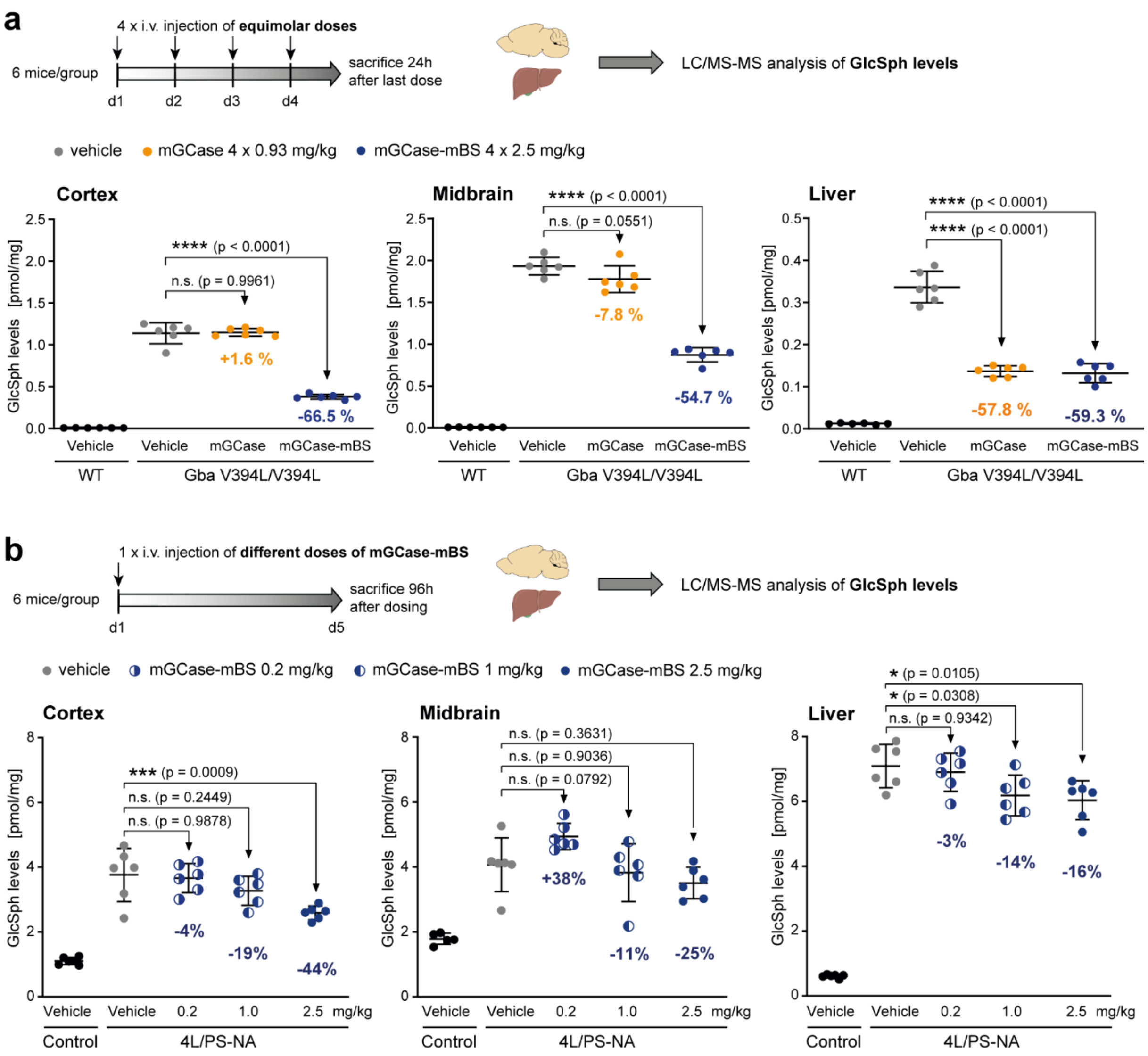

Supplementary Fig. 8. PD study in Gba V394L/V394L mice and dose-response study in 4L/PS-NA mice.

a Multi-dose PD study in Gba V394L/V394L mice to compare equimolar doses of mGCase vs. mGCase-mBS. GlcSph levels were measured in cortex, midbrain and liver. n = 6 mice/group. **b** Dose-response study in 4L/PS-NA mice to measure GlcSph levels in cortex, midbrain and liver samples at 96 h post single injection of 0.2 mg/kg, 1.0 mg/kg or 2.5 mg/kg of mGCase-mBS. n = 6 mice/group.

Data is represented as group mean +/- SEM. Data was analysed by one-way ANOVA (Dunnett's multiple comparisons test) comparing each treatment group to Gba V394L/V394L or 4L/PS-NA vehicle. n.s. $p > 0.05$; * $p < 0.05$; ** $p < 0.01$; *** $p < 0.001$; **** $p < 0.0001$. Schematics created with BioRender.com. Source data are provided as a Source Data file.

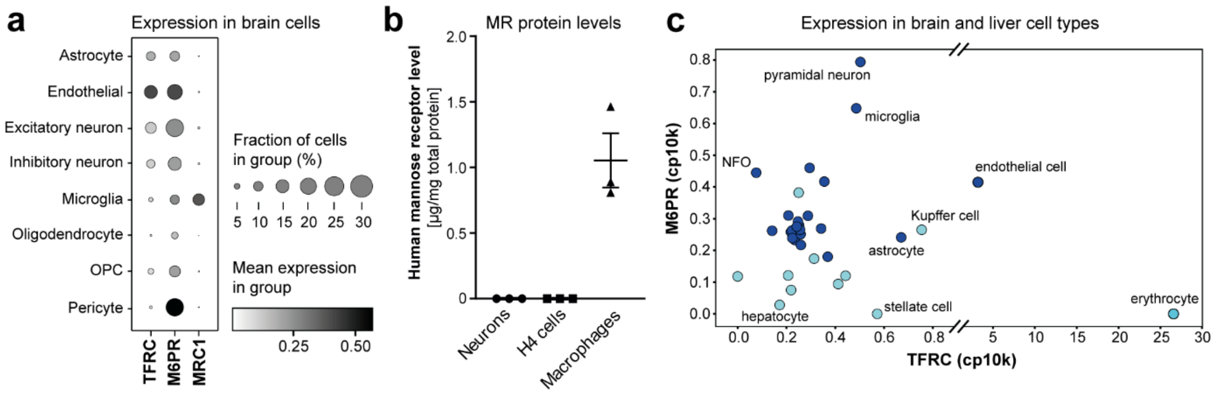

Supplementary Fig. 9. M6PR, TFRC and MRC1 expression levels in different cell types.

a Dot plot depicting the expression levels of TFRC, M6PR and MRC1 in different cell types of brain tissue. The white-to-black gradient reflects mean expression levels per group, dot size reflects fraction of cells per group that express TFRC, M6PR or MRC1. OPC: oligodendrocyte precursor cells. Source: ROSMAP data set[1]. **b** MR protein levels in human neurons, H4 cells or macrophages normalised to total protein. Error bars represent standard deviation. n = 3 independent measurements. **c** Dot plot illustrating the expression levels of M6PR and TFRC in different cell types of liver (turquoise dots) and brain (blue dots) tissue. NFO: newly formed oligodendrocyte. Source: human frontal cortex single nucleus transcriptome analysis[2]. Source data are provided as a Source Data file.

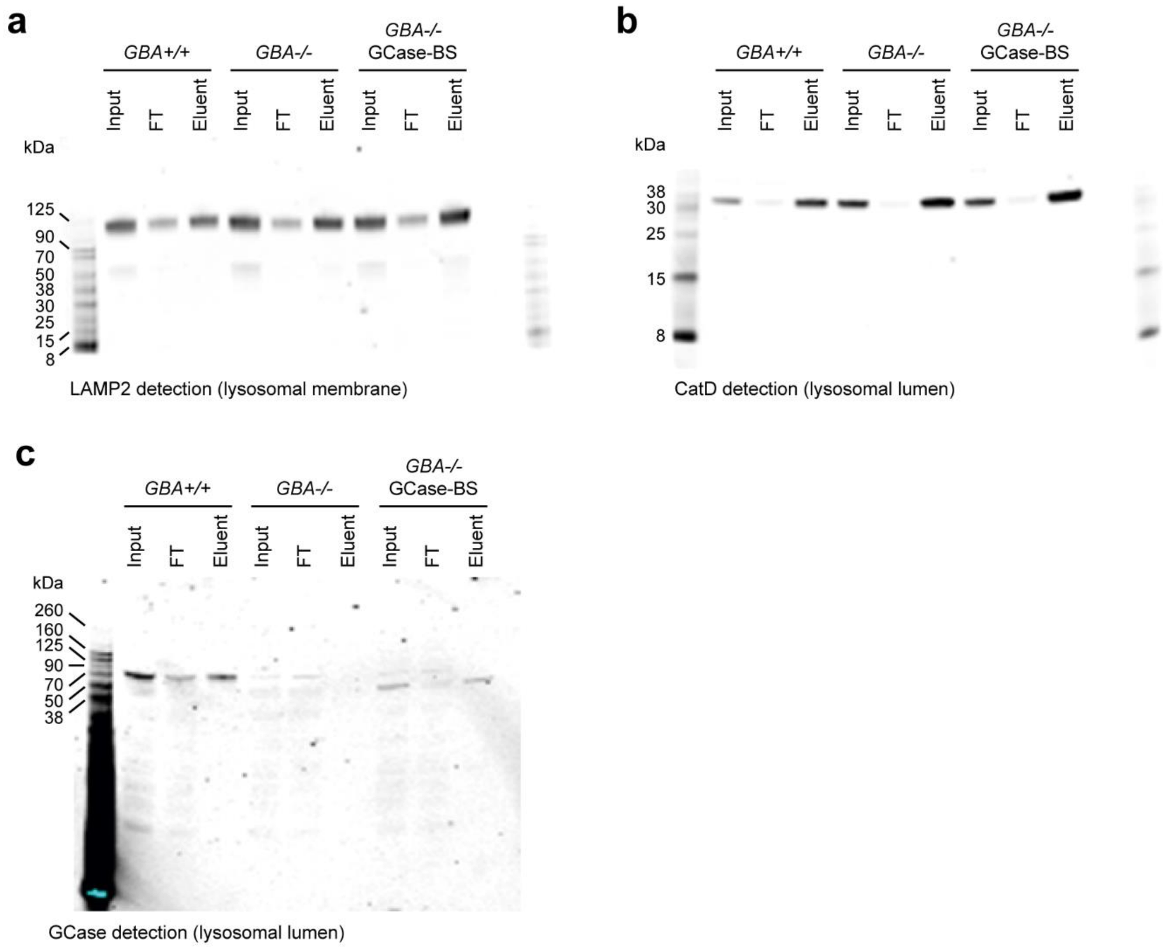

Supplementary Fig. 10. Uncropped blots of the Lyso-IP samples.

a Uncropped blot image of LAMP2 detection in the Lyso-IP samples of WT, GBA KO and GCase-BS treated GBA KO cells. **b** Uncropped blot image of CatD detection in the Lyso-IP samples of WT, GBA KO and GCase-BS treated GBA KO cells. **c** Uncropped blot image of GCase detection in the Lyso-IP samples of WT, GBA KO and GCase-BS treated GBA KO cells

Representative blots of 3 replicates. Molecular weight is indicated in kilodalton (kDa).

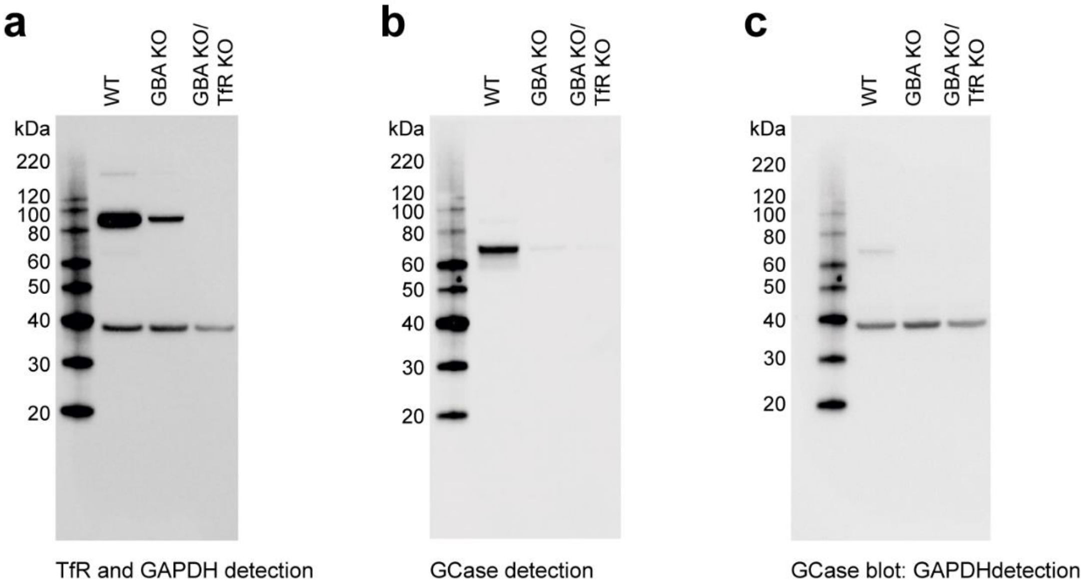

Supplementary Fig. 11. Uncropped blots of the validation of the GBA KO/TfR KO cell line.

a Uncropped blot image of TfR and GAPDH detection in WT, GBA KO and GBA KO/TfR KO cell lysates. **b** Uncropped blot image of GCase detection in WT, GBA KO and GBA KO/TfR KO cell lysates. **c** Uncropped image of the GCase blot after stripping and reprobing with GAPDH.

10 µg of total protein lysate were loaded per lane. Representative blots of 3 replicates. Molecular weight is indicated in kilodalton (kDa).

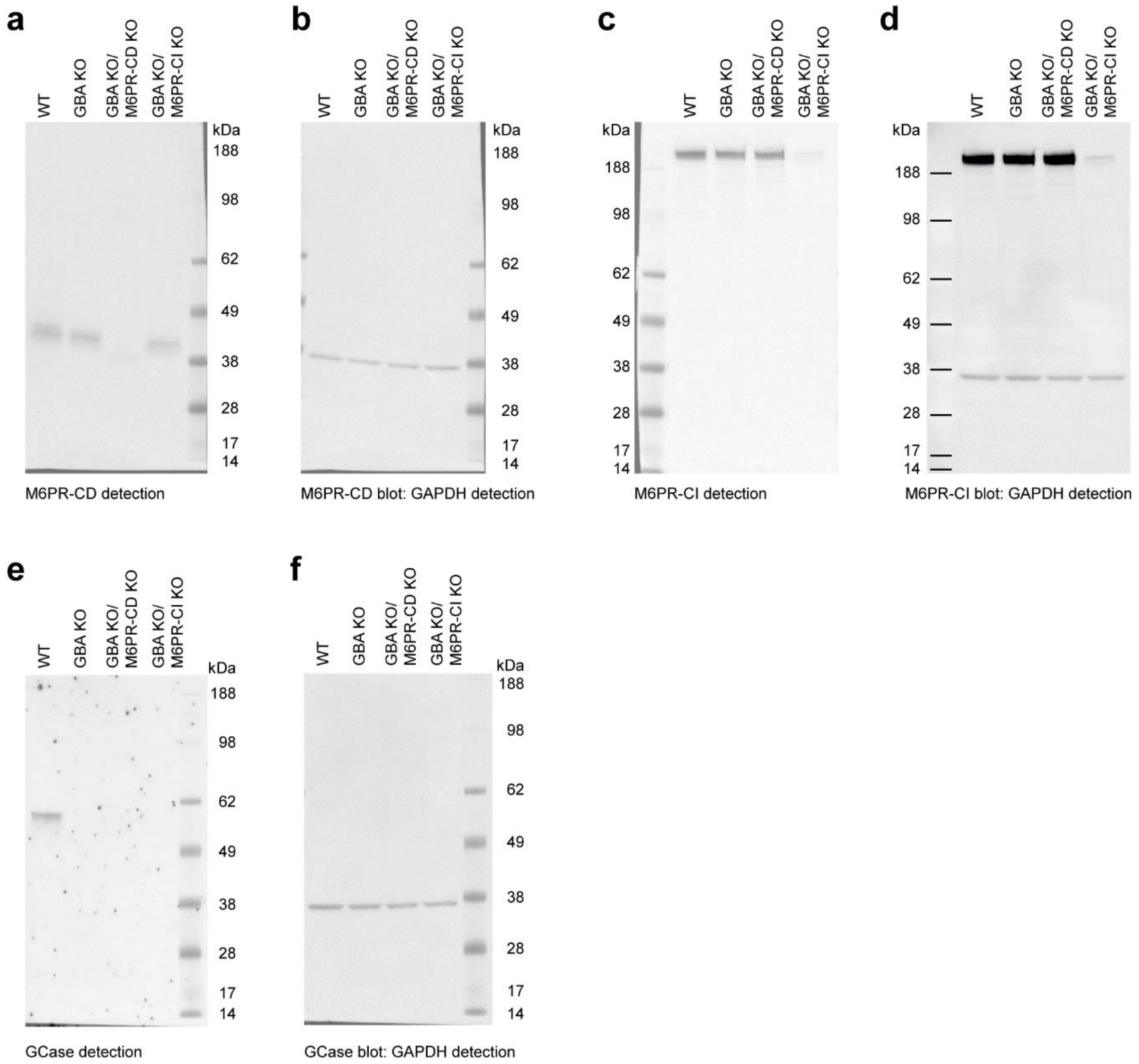

Supplementary Fig. 12. Uncropped blots of the validation of the GBA KO/M6PR KO cell lines.

a Uncropped blot image of M6PR-CD detection in WT, GBA KO and GBA KO/M6PR-CD KO and GBA KO/M6PR-CI KO cell lysates. **b** Uncropped image of the M6PR-CD blot after stripping and reprobing with GAPDH. **c** Uncropped blot image of M6PR-CI detection in WT, GBA KO and GBA KO/M6PR-CD KO and GBA KO/M6PR-CI KO cell lysates. **d** Uncropped image of the M6PR-CI blot after reprobing with GAPDH. **e** Uncropped blot image of GCase detection in WT, GBA KO and GBA KO/M6PR-CD KO and GBA KO/M6PR-CI KO cell lysates. **f** Uncropped image of the GCase blot after stripping and reprobing with GAPDH.

10 µg of total protein lysate were loaded per lane. Representative blots of 3 replicates. Molecular weight is indicated in kilodalton (kDa).

REFERENCES FOR SUPPLEMENTARY INFORMATION

1. Mathys, H. *et al.* Single-cell transcriptomic analysis of Alzheimer’s disease. *Nature* 570, 332–337 (2019).

2. Lau, S.-F., Cao, H., Fu, A. K. Y. & Ip, N. Y. Single-nucleus transcriptome analysis reveals dysregulation of angiogenic endothelial cells and neuroprotective glia in Alzheimer’s disease. *P Natl Acad Sci Usa* 117, 25800–25809 (2020).
