# Supplementary material for: Targeting neuronal lysosomal dysfunction caused by β-glucocerebrosidase deficiency with an enzyme-based brain shuttle construct
Source: Nat Commun. 2023 Apr 12;14:2057. doi: 10.1038/s41467-023-37632-4 (PMC10097658; doi:10.1038/s41467-023-37632-4)
Supplement: Supplementary file 2 — Description of Additional Supplementary Files [file 41467_2023_37632_MOESM2_ESM.pdf]

Description of Additional Supplementary Files

Supplementary Data 1: List of identified bona fide lysosomal proteins. Dataset enlisting identified bona fide lysosomal proteins in Lyso-IP samples (lysosomes). n = 3 biological replicates.

Supplementary Data 2: Statistics Proteomics and Lipidomics. Datasets showing metric changes for proteins or lipids respectively in GBA KO and GCase rescued samples. Datasets correspond to Fig. 5c-d and Supplementary Fig. 8a-b, respectively.

Supplementary Data 3: Lipidomics Raw Data compiled. Dataset comprising all relevant lipidomics data which is shown in Fig. 5d and Supplementary Fig. 8b.
